# A Monolithic 3D-Printed Platform for Functional Maturation and In Situ Contractility Assessment of 3D Skeletal Muscle

**DOI:** 10.34133/bmr.0363

**Published:** 2026-05-13

**Authors:** Jongwoo Ahn, Seonghun Mun, YoungWon Koo, Sangmin Lee, Seung-Hoon Yang, Seo Hyeon Choi, Jin Hong Mok, Jungho Ahn, Steve K. Cho, Jihoon Ko, Seokyoung Bang

**Affiliations:** ^1^ Department of Biomedical Engineering, Dongguk University, Goyang 10326, Republic of Korea.; ^2^ Department of Food Science and Biotechnology, Dongguk University, Goyang 10326, Republic of Korea.; ^3^ Department of MetaBioHealth, Sungkyunkwan University, Suwon 16419, Republic of Korea.; ^4^ Department of Biophysics, Institute of Quantum Biophysics, Sungkyunkwan University, Suwon 16419, Republic of Korea.; ^5^ Department of Biomedical Science and Engineering, College of Life Sciences and Medical Convergence, Gwangju Institute of Science and Technology (GIST), Gwangju 61005, Republic of Korea.; ^6^ Department of Life Sciences, College of Life Sciences and Medical Convergence, Gwangju Institute of Science and Technology (GIST), Gwangju 61005, Republic of Korea.; ^7^ Department of BioNano Technology, Gachon University, Seongnam 13120, Republic of Korea.

## Abstract

Sarcopenia—a debilitating consequence of global population aging characterized by the loss of muscle mass and function—demands in vitro platforms that enable a rigorous and quantitative assessment of muscle contractility. Pillar-displacement-based microphysiological systems are promising for this purpose but suffer from tension loss as tissues compact, creating variable boundary conditions and undermining reliability. We developed a monolithic, 3-dimensional printed Fast-Optimizing and Regeneration/Contraction-Evaluating platform featuring a detachable polydimethylsiloxane spacer that maintains a constant interpillar distance during long-term culture. The monolithic structure, fabricated by stereolithography, ensures high architectural reproducibility. Under the fixed-length boundary conditions, engineered muscles exhibited improved cellular alignment, enhanced myogenic differentiation, and more advanced structural maturation, resulting in markedly higher twitch and tetanic forces upon electrical stimulation. Together, these results establish the Fast-Optimizing and Regeneration/Contraction-Evaluating platform as a robust and reproducible muscle microphysiological system with fixed-length boundary conditions, enabling reliable, long-term quantitative evaluation of morphological and functional changes for tissue-engineering, drug-screening, and muscular-disease-modeling applications.

## Introduction

The rising global burden of population aging has established sarcopenia—a condition tightly linked to chronic disease and age-related decline—as a critical research priority with unmet medical needs [1]. Sarcopenia is a complex disorder defined by systemic reductions in skeletal muscle mass, strength, and function, underpinned by cellular and tissue-level pathologies including diminished satellite cell regenerative capacity, mitochondrial dysfunction, extracellular matrix remodeling, and reduced myofiber cross-sectional area [2]. To elucidate these mechanisms and to enable therapeutic screening, there is a substantial need for advanced in vitro models that faithfully recapitulate physiological contractile function. Such models are essential to overcome limitations of animal systems—namely, species-specific differences and reproducibility challenges—and conventional 2-dimensional cultures that fail to capture higher-order tissue functions [3].

Skeletal muscle exhibits a hierarchical architecture composed of parallel-aligned, multifunctional myofibers with repetitive sarcomere units [4]. Functionally, it displays pronounced plasticity—i.e., long-term adaptation to electrical and mechanical stimuli—and robust responsiveness to external cues [5]. However, achieving long-term organization and physiological maturation of myofibers in 3-dimensional (3D) hydrogel-based muscle models remains challenging, largely due to mechanical instability of the matrix and the nonuniform delivery of requisite stimuli [6,7]. Consequently, microphysiological systems are increasingly recognized as essential for accurately recapitulating complex muscle physiology in vitro [8].

Rigorous assessment of in vitro muscle maturation requires a shift from morphology or marker expression alone toward quantitative evaluation of actual contractile function [9]. Platforms that directly quantify force—such as microcantilevers [10,11], force transducers [12,13], and pillar-displacement measurement (PDM) systems [14–16]—have gained considerable traction. Among these, PDM platforms are attractive because they permit force readouts without complex external instrumentation. Critically, however, PDM systems often suffer from boundary-condition instability during long-term culture. Tissue compaction driven by intrinsic cellular contractility progressively reduces the interpillar distance, relaxing axial tension that should remain constant; this introduces variable boundary conditions that compromise reproducibility and reliability. The resulting spatially nonuniform tension further disrupts cellular alignment and blunts contractile responsiveness [17,18].

Establishing a fixed-length boundary condition that maintains the initial interpillar spacing is therefore imperative for long-term maturation and reliable functional evaluation. To control the mechanical environment in PDM platforms, 2 general strategies have been reported. First, externally actuated systems (e.g., magnetic/vacuum-based systems) can reversibly modulate mechanical resistance and provide in situ mechanical interrogation while preserving optical readouts [19,20]. Second, auxiliary-structure-based approaches (e.g., detachable braces or mold/holder-based workflows) can strengthen boundary conditions or limit changes in tissue length during maturation [21,22].

**Fig. 1.**
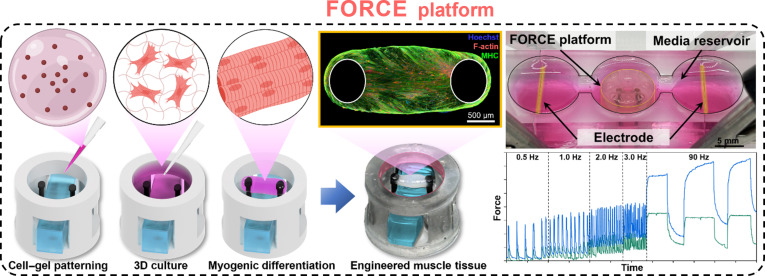
Overview of the Fast-Optimizing and Regeneration/Contraction-Evaluating (FORCE) platform with polydimethylsiloxane (PDMS) block spacer. A cell-laden hydrogel is patterned in an open suspended channel and, after 3-dimensional (3D) culture and myogenic differentiation, forms engineered muscle spanning force-sensing pillars while a removable PDMS block spacer maintains a constant interpillar distance. Representative immunofluorescence (Hoechst, F-actin, and myosin heavy chain [MHC]) shows aligned myofibers. A separate photograph shows a media-bridged reservoir and paired electrodes used to quantify contractility by electrical pulse stimulation (EPS). Real-time force traces during a stepwise frequency sweep (0.5 to 90 Hz) demonstrate frequency-dependent contractility.

Here, we introduce and validate a 3D-printed Fast-Optimizing and Regeneration/Contraction-Evaluating (FORCE) platform (Fig. 1). This system addresses tissue shortening and boundary-condition variability inherent to pillar-based platforms by maintaining a constant interpillar distance throughout long-term culture. The platform features a monolithic 3D-printed structure that integrates a detachable polydimethylsiloxane (PDMS) block spacer. For clarity, we refer to the spacer-installed condition state that fixes the interpillar distance as the “fixed-length boundary condition”. This design confers 2 principal advantages: (a) Single-process fabrication of the monolithic frame eliminates subtle intercomponent alignment errors, enhancing structural reproducibility [23]; and (b) the removable PDMS spacer mechanically constrains the interpillar distance during maturation to mitigate compaction-driven shortening and boundary-condition drift. The spacer can be removed prior to force acquisition so that compliant pillars remain available for sensitive displacement-based optical readout. Additionally, FORCE initiates tissue formation in an open/suspended geometry, and tissue attachment is consistently observed near the pillar free ends. This can reduce ambiguity in the effective attachment point for displacement-based force quantification. Collectively, FORCE improves structural stability and alignment reproducibility and provides a muscle microphysiological system with fixed-length boundary conditions suitable for quantitative evaluation of morphological and functional changes during long-term culture.

## Materials and Methods

### Cell culture

Mouse myoblasts (C2C12; American Type Culture Collection, USA) were used to generate 3D muscle constructs. Cells between passages 2 and 5 were cultured in growth medium (Dulbecco’s modified Eagle's medium, high glucose, supplemented with 10% fetal bovine serum and 1% penicillin–streptomycin) and maintained at 37 °C in a humidified incubator with 5% CO_2_. Cells were subcultured at 60% to 70% confluency using 0.25% trypsin–EDTA (Gibco, USA), and the growth medium was replaced every other day.

### Design and fabrication of the FORCE platform

The FORCE platform was fabricated using a stereolithography (SLA) 3D printer (Form 3; Formlabs Inc., USA) with Elastic 50A resin (Elastic 50A resin V2; Formlabs Inc.). Computer-aided designs were created using Fusion 360 (Autodesk, USA), and printing was performed at a layer thickness of 100 μm. After printing, the samples were washed in isopropyl alcohol for 20 min and postcured under ultraviolet (UV) light at 70 °C for 30 min, following the manufacturer’s protocol.

To enhance biocompatibility and prevent resin leaching, the printed structures were coated with a 0.5-μm-thick parylene C layer (OBT-PC300; Obang Technology, Korea) using chemical vapor deposition. The coated platforms were sterilized by UV exposure for 30 min prior to cell seeding.

The PDMS block spacer was fabricated by mixing the Sylgard 184 (Dow Corning, USA) base and curing agent at a 10:1 (w/w) ratio. The mixture was poured into a 3D-printed mold, precoated with 1-μm-thick parylene C, then homogenized, degassed, and cured at 80 °C for 1 h. After demolding, the spacer was placed between the micropillars within the suspended channel during tissue culture to maintain the initial interpillar distance.

### Seeding C2C12 in the FORCE platform

The cells were detached using 0.25% trypsin–EDTA (Gibco) and collected by centrifugation. The harvested cells were resuspended at a final density of 6 × 10^6^ cells/ml and mixed on ice with a pre-neutralized collagen–Matrigel solution composed of rat tail collagen type I (Corning, USA), Matrigel (Corning), 10× phosphate-buffered saline (PBS), and 1× PBS to prepare the cell–gel hydrogel mixture. The final concentrations of collagen type I and Matrigel in the hydrogel were 2 mg/ml and 0.82 mg/ml, respectively, based on previously reported formulations [24].

A total of 30 μl of the prepared cell–gel hydrogel was carefully injected into the suspended channel. The loaded device was incubated at 37 °C for 30 min to allow complete gelation. After gelation, the platform was inverted and cultured in a 48-well plate. After 2 d of culture, the medium was replaced with differentiation medium (Dulbecco’s modified Eagle's medium, high glucose, supplemented with 2% horse serum, 1% penicillin–streptomycin, and 50 ng/ml insulin-like growth factor-1) to induce myogenic differentiation. The culture medium was changed every other day throughout the culture period.

### Live/dead assay

To evaluate the cytocompatibility of the parylene C coating, cell viability was assessed on parylene C-coated (0.5 μm) and uncoated devices using the Live/Dead Viability/Cytotoxicity Kit for Mammalian Cells (L3224; Thermo Fisher Scientific, USA) according to the manufacturer’s protocol. The devices were UV sterilized for 30 min and placed in a 48-well plate, and C2C12 cells were seeded on the bottom surface. Cell viability was examined at 24, 48, and 72 h after seeding. In addition, Hoechst 33342 (Thermo Fisher Scientific) was used for nuclear staining to quantify total cell numbers. Briefly, the culture medium was removed, and the samples were washed twice with PBS (pH 7.4). A staining solution containing 2 μM calcein acetoxymethyl ester, 4 μM ethidium homodimer-1, and 5 μg/ml Hoechst 33342 in PBS was freshly prepared. The samples were incubated with the solution at 37 °C for 30 min in the dark, washed once with PBS, and imaged using a fluorescence microscope (LS40; LEAM, Korea). Cell viability (%) was calculated as the number of calcein-positive cells divided by the total number of Hoechst-stained nuclei within the region of interest.

### Immunocytochemistry

Skeletal muscle tissues were fixed with 4% paraformaldehyde (GeneAll Biotechnology, Korea) for 30 min at room temperature (RT) and rinsed twice with PBS. Fixed samples were permeabilized and blocked in a blocking buffer containing 1% bovine serum albumin, 2% serum, and 0.2% Triton X-100 in PBS for 1 h at RT. After blocking, tissues were incubated overnight at 4 °C with a primary antibody against myosin heavy chain (MHC, PA5-115216, Thermo Fisher Scientific; 1:200 dilution). Following primary incubation, samples were washed 3 times with PBS and then incubated for 2 h at RT with the secondary antibody, goat anti-rabbit immunoglobulin G (H+L) cross-adsorbed secondary antibody conjugated with Alexa Fluor 488 (A-11008, Thermo Fisher Scientific; 1:1,000 dilution). Actin filaments were subsequently stained using an F-actin staining kit (ab112127; Abcam, UK) for 2 h at RT. Nuclear counterstaining was performed with Hoechst 33342 (H3570, Thermo Fisher Scientific; 1:2,000 dilution) for 30 min at RT. All antibody and staining solutions were prepared using the same blocking buffer described above.

### Analysis of morphology

Distance between 2 force-sensing pillars spanned by each tissue construct was measured. Bright-field images were captured every other day using an inverted microscope (LS40; LEAM). The interpillar distance was determined by calculating the Euclidean distance between the centroids of the 2 pillars. All image analyses were performed under identical settings using Fiji (ImageJ; NIH, USA), and the measured values were used directly without normalization.

To evaluate the cellular alignment of engineered muscle tissues, F-actin-stained samples were imaged using a confocal fluorescence microscope (K1-Fluo; Nanoscope Systems, Korea). For each construct, 3 nonoverlapping regions within the central span between pillars were randomly selected for analysis.

Directionality analysis was performed in Fiji (ImageJ; NIH) using the Directionality plugin based on a Fourier-transform algorithm. Orientation histograms were generated over an angular range from −90° to +90° with a bin size of 5°, and the distribution *P*(*θ*) of actin fiber orientations was extracted. The orientation index (OI) was defined as the cosine-weighted average of the angular distribution, expressed as (Eq. 1)

$$\mathrm{OI}=\sum_{i=1}^{K}P\left(\theta_{i}\right)\cos\left(\theta_{i}\right),\theta_{i}\in \left[-90{}^{\circ},\;90{}^{\circ}\right]$$ (1)

where $P\left(\theta_{i}\right)$ represents the normalized frequency at each angle *θ* and *K* denotes the number of angle bins spanning −90° to +90° (bin size: 5°; *K* = 36). This approach emphasized horizontal alignment: Vertically aligned fibers yielded an OI of 0, horizontally aligned fibers yielded an OI of 1, and an isotropic orientation yielded an OI of 2/π (≈0.637). All analyses were performed using identical parameter settings to ensure reproducibility.

### Force–displacement test and modulus estimation

The sample was measured using a texture analyzer (TA.XTplus; Stable Micro Systems, Surrey, UK) in compression mode. The sample was fixed with the lower part of the Tensile Grips (A/TG), and a Blade Set knife probe (HDP/BS-B; width 7.0 cm) was used. The probe was positioned 23.3 mm from the sample’s fixed axis, moved vertically downward by 10.00 mm, and then returned to the initial position. Test speeds were set to 1.00 mm/s (pretest), 1.00 mm/s (test), and 10.00 mm/s (posttest). All measurements were performed in triplicate at the same location. From 3 tests, we obtained a stiffness of *k* = 4.42 ± 1.00 μN/μm (*n* = 3) and, by Euler–Bernoulli back-calculation, a material modulus of *E* = 2.414 MPa. These values were used in the “Finite-element analysis: pillar deformation” and “Analysis of contractile force” sections.

### Finite-element analysis: pillar deformation

A 3D finite-element model of the micropillar was developed using COMSOL Multiphysics (COMSOL Inc., Sweden) with the Solid Mechanics module under stationary conditions and with geometric nonlinearity enabled. The pillar material was modeled as a nearly incompressible elastic solid with a Young’s modulus (*E*) of 2.414 MPa and a Poisson’s ratio (*ν*) of 0.49. The pillar base was fully constrained to represent rigid attachment to the substrate. To simulate the traction generated by the engineered muscle tissue, a lateral load was applied to the top region (*L*) of the pillar using the Total Force boundary condition, where a prescribed total force (*F*_pillar_) was uniformly distributed across the 2 opposite side faces. Following a previous report [25], a static contraction force of 76 μN (corresponding to 38 μN per pillar in a 2-pillar configuration) was used as the reference load. This value was employed to examine structural conditions that allow the pillars to respond sensitively to small contractile changes while avoiding excessive approach under static tension. All simulations were performed using a nonlinear stationary solver with line search enabled. To ensure convergence, an auxiliary ramp parameter (*s* ∈ [0, 1]) was introduced to gradually scale the applied load (*t* = *s* · *t*_0_). The mesh was locally refined around the loaded region to improve computational accuracy.

### Finite-element analysis: electric field distribution

Finite-element analysis (FEA) of the electric field was conducted using COMSOL Multiphysics (COMSOL Inc.) with the Electric Currents module under stationary conditions. A potential difference of 40 V was applied between 2 electrodes positioned 22 mm apart, corresponding to a theoretical field strength of approximately 1.82 V/mm by simple calculation. The simulation showed that the electric field in the region containing the tissue-engineered muscle ranged from 1.1 to 1.7 V/mm, slightly lower than the theoretical estimation. Minor variations in field intensity were observed around the micropillars, primarily due to the insulating properties of the parylene C coating on the resin surface, which resulted in locally higher (>2.0 V/mm) or lower (<1.0 V/mm) regions. Overall, the simulated field distribution was consistent with previously reported effective and nondamaging ranges for in vitro muscle stimulation [26,27].

### Analysis of contractile force

The contractile force generated by the engineered muscle tissues was quantified from pillar-deflection measurements using a combined beam-theory and FEA framework. Micropillars were fabricated by 3D printing with elastic resin with an elliptical cross-section (major semi-axis *a* and minor semi-axis *b*) and a total height *h* (typically *a* = 300 μm, *b* = 200 μm, and *h* = 4 mm). The mechanical behavior of the pillar material was modeled using a nearly incompressible Neo-Hookean formulation, where the Young’s modulus (*E*) was set to 2.414 MPa and the Poisson’s ratio (*ν*) was set to 0.49. These values were based on literature data [28] and verified by FEA and beam theory, yielding a shear modulus of approximately *μ* ≈ 0.81 MPa. Pillars were modeled as cantilever beams subjected to lateral loading. For a lateral force applied at a height *h* above the base, the lateral stiffness *k*(*h*) was calculated using the Euler–Bernoulli beam relation (Eq. 2):

kh=3EIh3 (2)

where *I* is the second moment of inertia for an elliptical cross-section (Eq. 3):

I=πab34 (3)

where *a* and *b* are the semi-major and semi-minor axes, respectively, and *b* corresponds to the semi-axis along the bending direction. The tissue-generated force (*F*) was then determined using Hooke’s law (Eq. 4):

$$F=k\left(h\right)\delta$$ (4)

where *δ* represents the lateral deflection of a single pillar measured at height *h*.

Prior to contractility measurements, the PDMS block spacer was removed under submerged conditions to minimize mechanical disturbance to the tissues (Movie S1). The FORCE platform was gently retrieved from the 48-well plate using forceps and transferred to a dish filled with culture medium so that the entire device remained fully submerged during handling. The device was oriented to expose both ends of the spacer. Using a second forceps, the rigid region of the device distant from the micropillars and tissue was lightly stabilized while avoiding contact with the pillars or tissue. One end of the PDMS block spacer was then grasped and removed by pulling it straight along the spacer’s longitudinal axis at a slow and constant speed to prevent sudden jerking. Immediately after removal, tissues were inspected to confirm continuous attachment to both pillars without tearing or partial detachment. The device was then transferred to the 3-well stimulation platform filled with medium for electrical stimulation and video-based pillar-deflection analysis.

Electrical stimulation was applied using a Myopacer field stimulator (IonOptix, USA) to induce periodic muscle contractions. Twitching force measurements were performed at 1 Hz with a 10-ms pulse width, while tetanic force measurements were conducted at 90 Hz with a 10-ms pulse width. Muscle contraction videos were recorded at 20× magnification using an inverted optical microscope (LS40; LEAM). Pillar deflections were tracked frame-by-frame using the open-source software Tracker (Open-Source Physics, USA), and displacement values were calibrated according to the microscope’s pixel-to-length conversion.

### Pharmacological treatment

Engineered muscle tissues were cultured and differentiated on the FORCE platform until day 7. To probe pharmacological responsiveness, constructs were exposed to dexamethasone (DEX; Fujifilm, Japan) or testosterone (TES; Fujifilm, Japan) for 48 h. DEX and TES were first dissolved in dimethyl sulfoxide (DMSO; Sigma-Aldrich, USA) to prepare stock solutions and then diluted in differentiation medium immediately before treatment. Final treatment concentrations were 0.01, 0.1, and 1.0 mM for DEX and 0.01, 0.1, and 1.0 μM for TES. Vehicle controls received an equivalent volume of DMSO, and the final DMSO concentration was matched across all groups. After 48 h of exposure, twitch and tetanic contractile forces were quantified using pillar-deflection-based analysis as described in the “Analysis of contractile force” section.

### Statistical analysis

All statistical analyses were performed using GraphPad Prism software (version 9.0; GraphPad Software, USA). For comparisons between 2 groups, an unpaired 2-tailed *t* test was applied. Differences among multiple groups were analyzed using 1-way or 2-way analysis of variance followed by Tukey’s multiple comparisons post hoc test, as appropriate. The significance threshold was set at *P* <0.05. Statistical significance is denoted as follows: *P < 0.05; **P < 0.01; ***P < 0.001; ****P < 0.0001; ns, not significant. Data are presented as mean ± standard deviation, unless otherwise stated. In addition to *P* values, effect sizes (Cohen’s *d* and *η*^2^) were reported. A full statistical summary is provided in Tables S1 to S3.

## Results and Discussion

### A PDMS block-supplemented FORCE platform

**Fig. 2.**
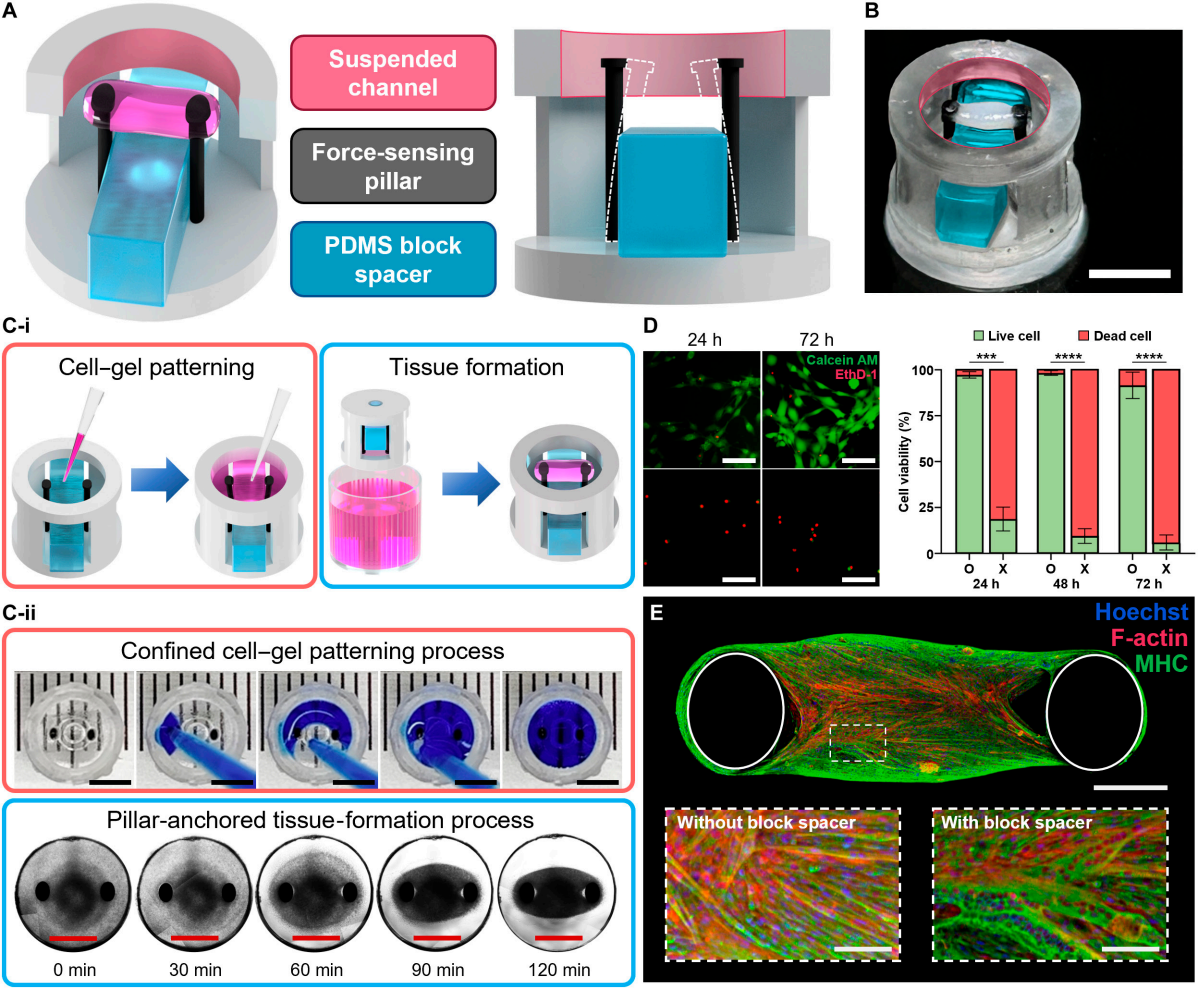
Structural design of the FORCE platform and formation of matured muscle tissue. (A) Experimental illustration of the FORCE platform comprising a suspended hydrogel channel (pink), elastic force-sensing pillars (black), and a removable PDMS spacer (blue). (B) Bright-field image of engineered muscle following self-assembly and compaction of the patterned cell-laden hydrogel within the suspended channel. Scale bar, 3 mm. (C-i) Experimental workflow: patterning of a cell-laden hydrogel within the channel (red box), followed by tissue formation via self-assembly after gelation (blue box). (C-ii) Demonstration of gel patterning using blue dye (red box) and time-lapse images showing cell-driven contraction and rearrangement over 120 min (blue box). Scale bar, 3 mm. (D) Effect of parylene C coating on cell viability. Live/dead images (calcein acetoxymethyl ester [Calcein AM], green; ethidium homodimer-1 [EthD-1], red) with quantitative analysis (*n* = 5). Asterisks in panel D indicate ***P < 0.001 and ****P < 0.0001. Scale bar, 100 μm. (E) Immunofluorescence of engineered muscle on the FORCE platform (nuclei, Hoechst, blue; F-actin, red; MHC, green). Insets: confocal comparison of myofiber morphology with and without the PDMS spacer. Scale bars, main 500 μm; insets 100 μm.

We developed the FORCE platform as a monolithic structure comprising a suspended channel and a force-sensing pillar set, supplemented with a PDMS block spacer to recapitulate and evaluate engineered skeletal muscle tissue (Fig. 2A and B). To initiate 3D tissue formation, a cell-laden hydrogel was dispensed along the wall of the open suspended channel and, via surface tension, filled the central region (Fig. 2C-i). Immediately after gelation, the device was transferred into the culture medium, where cell-driven compaction induced hydrogel contraction and generated a 3D muscle construct spanning the 2 pillars (Fig. 2C-ii). Because SLA resins can leach cytotoxic species during long-term culture, we deposited a parylene barrier coating on the 3D-printed resin prior to cell and hydrogel patterning. Parylene improved cell viability relative to uncoated devices (Fig. 2D) and is a durable biointerface that mitigates leachables during culture [29]. Within the FORCE platform, the PDMS block spacer was associated with enhanced myogenic differentiation, reflected by increased MHC expression and thicker, fused myofibers, compared with FORCE devices operated without the spacer (Fig. 2E).

The elastic, force-sensing pillars support in vitro 3D muscle formation and enable convenient quantification of contractile forces, an approach widely used in muscle research [30]. In FORCE, the pillars and suspended channel were printed as a single monolithic device in one SLA fabrication step. Each unit was sized to the footprint of a 48-well plate well, enabling higher-throughput tissue fabrication and scalable functional readouts in a standard multiwell format. The PDMS block spacer was assembled between pillars to impose a fixed-length boundary condition during tissue formation and differentiation and was subsequently removed to enable displacement-based force measurements. This approach allowed culture and measurement conditions to be independently controlled while preserving tissue integrity. Collectively, the 3D-printed, parylene-coated FORCE platform retains the advantages of pillar-based muscle-on-chip systems and supports robust muscle formation, differentiation, and functional analysis.

### Optimization of 3D-printed force-sensing pillar dimensions in the FORCE platform

**Fig. 3.**
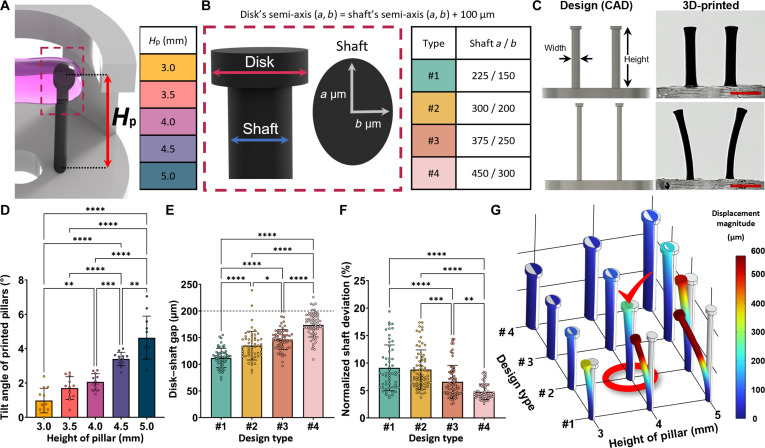
Optimization and dimensional analysis of 3D-printed force-sensing pillars for the FORCE platform. (A) Experimental design defining pillar height (*H*_p_). (B) Pillar architecture with elliptical shaft and disk; disk height, 200 μm, and disk semi-axes each 100 μm larger than the corresponding shaft semi-axes. (C) Computer-aided design (CAD) models and corresponding prints illustrating structural fidelity for 2 pillar geometries. Scale bar, 2 mm. (D) Tilt (bending) angle of printed pillars as a function of *H*_p_ (*n* = 12). (E) Disk–shaft gap measured across design variants (*n* ≥ 25). (F) Normalized deviation of printed shaft thickness quantified from side-view images (*n* ≥ 25). Asterisks in panels D to F indicate *P < 0.05, **P < 0.01, ***P < 0.001, and ****P < 0.0001. (G) Finite-element analysis (FEA) of pillar-tip displacement under a reference static contractile load (38 μN), comparing pillar designs and heights.

We next optimized force-sensing pillar geometry to balance displacement sensitivity with fabrication fidelity and reproducibility. Pillars were designed with a shaft–disk configuration to anchor tissue at the pillar tip; both shaft and disk had elliptical cross-sections (Fig. 3A and B). Pillar height (*H*_p_) was set to 3.0 to 5.0 mm (0.5-mm steps). The shaft used a major-to-minor axis ratio of 1.5:1; the minor axis (thickness) varied from 150 to 300 μm in 50-μm steps. The disk major and minor axes were each 100 μm larger than those of the shaft, with a disk height of 200 μm. Shorter and thicker pillars reproduced the intended geometry more faithfully, whereas taller or thinner pillars exhibited deviations including bending (Fig. 3C). The bending angle increased with height (Fig. 3D). Increasing thickness produced disk–shaft gaps closer to the designed 200 μm (Fig. 3E) and reduced thickness deviations (Fig. 3F). Finite-element simulations indicated that taller, thinner pillars yield larger deflections under contractile loading (Fig. 3G), improving displacement readout sensitivity, whereas shorter, thicker pillars produce displacements too small for high-resolution measurements.

To bias bending toward the contraction axis, FORCE employs elliptical pillars elongated in the direction orthogonal to the muscle bundle, which bend preferentially along the pillar line of action compared with circular pillars [20]. Given that larger center-to-center spacing can increase tensile loading and promote alignment and differentiation [31], we fixed the pillar spacing at 2.6 mm, the longest feasible span within the 48-well-scaled chip. To prevent tissue detachment, we used a split disk–shaft design [6,14,32]; although the disk feature was not perfectly replicated in all prints, no detachment occurred during subsequent culture.

Overall, pillar height and thickness exerted opposing effects: increasing *H*_p_ and decreasing thickness improved displacement sensitivity but reduced fabrication stability; decreasing *H*_p_ and increasing thickness improved manufacturability but reduced sensitivity. Because high aspect ratios increase susceptibility to lateral deformation—which can introduce force-inference errors from interpillar distance changes—geometry must balance robustness and resolvable displacement. We selected an intermediate geometry (major axis *a* = 300 μm, minor axis *b* = 200 μm, and *H*_p_ = 4 mm) for subsequent experiments.

### Optimization of suspended channel height

**Fig. 4.**
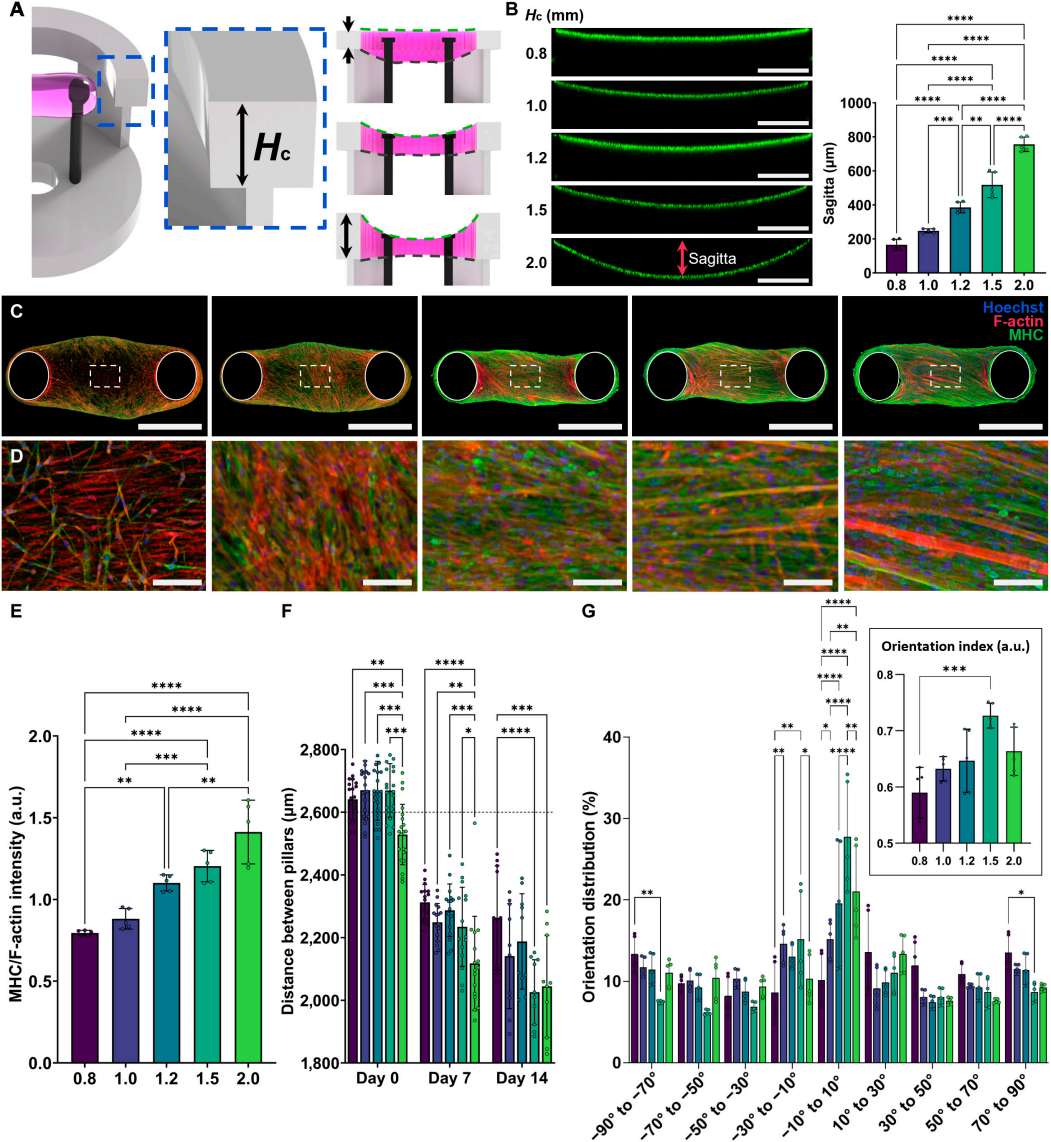
Effect of suspended channel height (*H*_c_) on hydrogel geometry and muscle tissue maturation. (A) Experimental design showing patterned hydrogel geometry as a function of *H*_c_, with sagitta (surface depth) indicated. (B) Fluorescence images (green tracer beads) and quantitative analysis of surface curvature and sagitta versus *H*_c_ (*n* ≥ 5). Scale bar, 1 mm. (C) Immunofluorescence of engineered muscle after 14 d under the indicated *H*_c_ conditions (nuclei, Hoechst, blue; F-actin, red; MHC, green). Scale bar, 1 mm. (D) Higher-magnification confocal images of boxed regions in (C). Scale bar, 100 μm. (E) Relative MHC fluorescence normalized to F-actin (*n* = 5). (F) Interpillar distance reduction (μm) over 14 d of culture (*n* ≥ 10). (G) Myofiber orientation distributions and orientation index (OI; see Methods) at day 14 as a function of *H*_c_ (*n* = 5). Asterisks in panels B and E to G indicate *P < 0.05, **P < 0.01, ***P < 0.001, and ****P < 0.0001.

Suspended-channel geometry co-defines hydrogel shape and thus the emergent tissue architecture. We varied channel height (*H*_c_) systematically (0.8, 1.0, 1.2, 1.5, and 2.0 mm), keeping a circular planform matched to a 48-well footprint (Fig. 4A). Immediately after loading 30 μl of fluorescent-bead-laden hydrogel, surface profiling showed that increasing *H*_c_ elevated the surface curvature and increased the sagitta (depth of the most concave region; Fig. 4B). The opposite hydrogel surface was also retained more stably at higher *H*_c_, whereas low *H*_c_ (0.8 to 1.0 mm) caused the same-volume gel to bulge beneath the channel along the pillars (Fig. S1). These loading differences translated to distinct postcompaction morphologies: tissue thickness (lateral view) varied with *H*_c_, with a statistically significant thickness reduction observed only at *H*_c_ = 1.5 mm over days 7 to 14 (Fig. S2). After 14 d of culture with C2C12-laden hydrogel, 3D constructs differed in morphology and in myogenic differentiation (Fig. 4C and D). Quantitatively, increasing *H*_c_ correlated with higher MHC expression (Fig. 4E). Using interpillar distance as a proxy for passive tension, the largest distance reductions were observed for *H*_c_ = 1.5 mm during both days 0 to 7 (1.5 mm: 434.9 ± 147.6 μm; 2.0 mm: 411.3 ± 173.1 μm) and days 7 to 14 (1.5 mm: 643.5 ± 124.4 μm; 2.0 mm: 484.7 ± 173.7 μm) (Fig. 4F and Fig. S3). Alignment along the pillar axis did not differ significantly by day 7, but *H*_c_ = 1.5 mm yielded the highest alignment by day 14 (Fig. 4G and Fig. S4).

At *H*_c_ = 0.8 to 1.0 mm, the hydrogel loaded thickly beneath the channel along the pillars, yielding relatively lower central alignment parallel to the interpillar axis—likely because compaction-driven tension dominated over pillar-imposed axial tension across a larger gel volume. At *H*_c_ ≥1.2 mm, the hydrogel remained confined within the channel and cells preferentially anchored to pillar tips, producing superior alignment along the designed axis. This is consistent with reports that 3D patterning geometry (e.g., support width) regulates alignment directionality [33]. We infer that spatial tension distributions during in-channel gelation and early compaction reorganize the hydrogel microstructure, governing alignment. Thus, hydrogel thickness is a critical determinant of myocyte alignment and tissue organization; here, higher *H*_c_ (thinner gels) promoted alignment parallel to the interpillar axis.

Notably, despite higher MHC at *H*_c_ = 2.0 mm, the *H*_c_ = 1.5 mm condition produced greater interpillar shortening and alignment by day 14. We attribute the 2.0-mm phenotype to its more concave loading geometry, which promoted early compaction and initial shortening at day 0, elevating passive tension during days 0 to 7 and potentially activating focal adhesion kinase–phosphatidylinositol 3-kinase–protein kinase B and mitogen-activated protein kinase signaling to enhance MHC expression [27]. From days 7 to 14, contraction in the 2.0-mm group plateaued, whereas continued shortening in the 1.5-mm group—supported by superior alignment and more uniform cell distribution—led to higher passive force. These data suggest that sustained passive-force enhancement is driven predominantly by structural integrity (alignment, uniformity) rather than marker expression alone, and that increased passive tension from optimized microstructure translates to greater active force generation [34]. We therefore used *H*_c_ = 1.5 mm for subsequent studies.

### Effects of PDMS block spacer on muscle-tissue formation

**Fig. 5.**
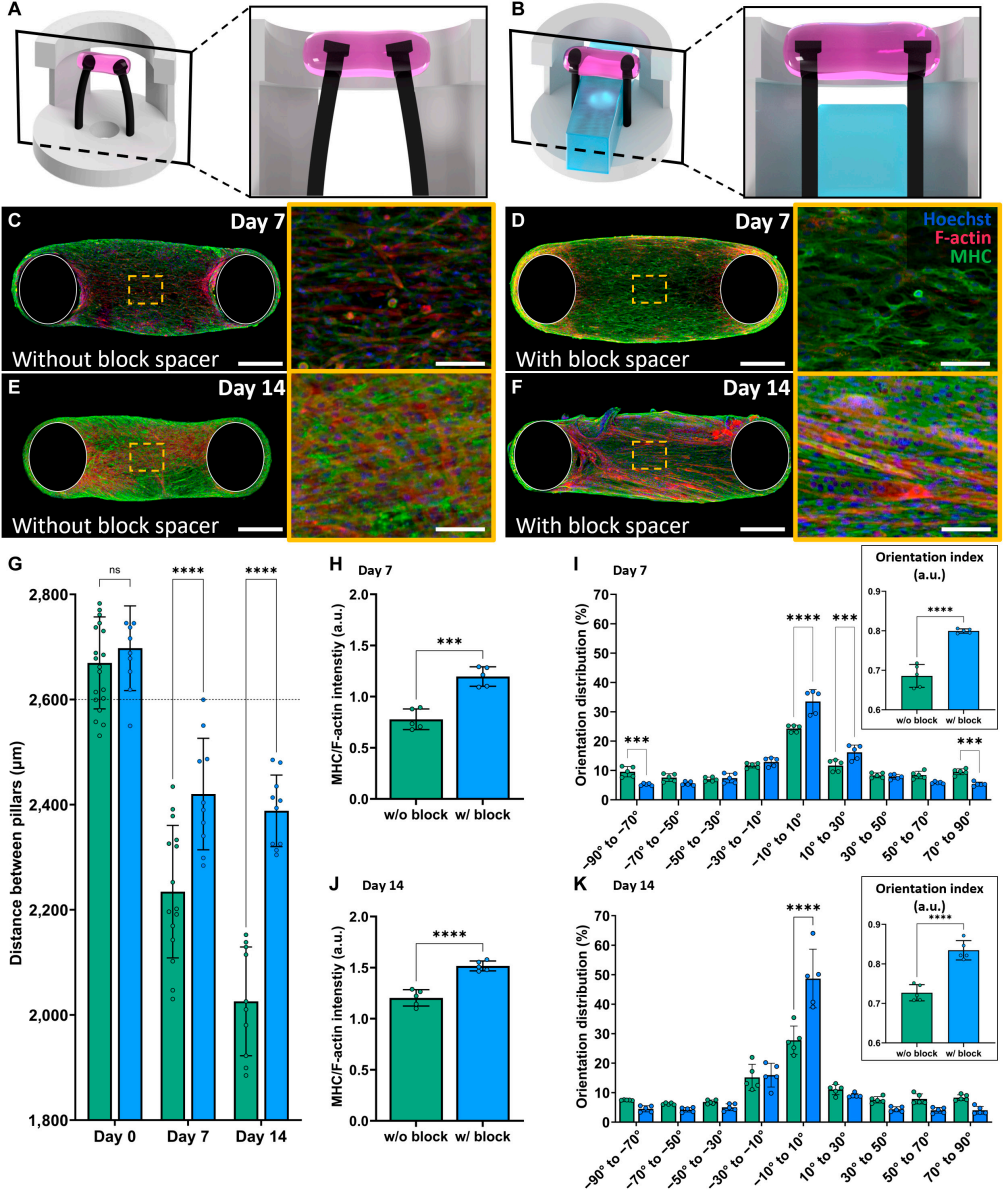
Enhanced muscle tissue morphology and maturation driven by the PDMS block spacer. (A and B) Experimental illustration of pillar-displacement dynamics. (A) Without the PDMS spacer, tissue compaction induces inward pillar deflection and interpillar shortening. (B) With the spacer, interpillar distance is maintained, enforcing fixed-length boundary conditions. (C and D) Day 7 immunofluorescence images (nuclei, Hoechst, blue; F-actin, red; MHC, green). Scale bars, main 500 μm; insets 100 μm. (E and F) Day 14 immunofluorescence images under the indicated conditions. Note increased myofiber thickness, alignment, and multinucleation with the PDMS spacer (F). Scale bars, main 500 μm; insets 100 μm. (G) Interpillar distance change (μm) over 14 d of culture (*n* ≥ 10). (H and J) Quantification of MHC fluorescence normalized to F-actin at day 7 (H) and day 14 (J) (*n* = 5). (I and K) Myofiber orientation distributions and OI at day 7 (I) and day 14 (K) (*n* = 5). Asterisks and statistical annotations in panels G to K indicate ns, not significant, ***P < 0.001, and ****P < 0.0001.

To prevent compaction-driven interpillar shortening during culture and thereby stabilize a fixed-length boundary condition, we inserted a PDMS block spacer between the pillars during maturation. This yielded stable 3D tissues. Compared with constructs cultured without the spacer (Fig. 5A, C, and E), tissues cultured with the spacer exhibited significantly higher MHC expression at days 7 and 14 (Fig. 5B, D, F, H, and J) and showed significantly improved cellular alignment at both time points (Fig. 5I and K). As expected, the spacer effectively prevented interpillar distance reduction despite passive contractile forces (Fig. 5G).

We posit that fixed-length (isometric) loading against the spacer reinforced extracellular matrix cross-linking and intracellular tensile structures (stress fibers), preserving alignment and facilitating passive-force accumulation [35–37]. Furthermore, a highly organized, unidirectional alignment of muscle fibers can enhance the efficiency of contractile-force transmission and accelerate structural maturation. While rigid pillars can also maintain a fixed-length boundary condition, we deliberately combined elastic pillars with a removable spacer to (a) enforce a constant interpillar distance during maturation, mitigating compaction-driven shortening and boundary-condition drift, and (b) enable seamless contractility measurements by removing the spacer without detaching tissue. This avoids transfer to external transducers, which risks damage and sample loss [38]. Elastic pillars alone enable displacement-based readouts but allow compaction-driven shortening during culture, limiting myogenesis and maturation [6,14,16,32,39]. Collectively, the spacer-supplemented FORCE platform couples fixed-length maturation with sensitive pillar-deflection readout, thereby promoting differentiation.

### Impact of the PDMS block spacer on contractility and tissue stability

**Fig. 6.**
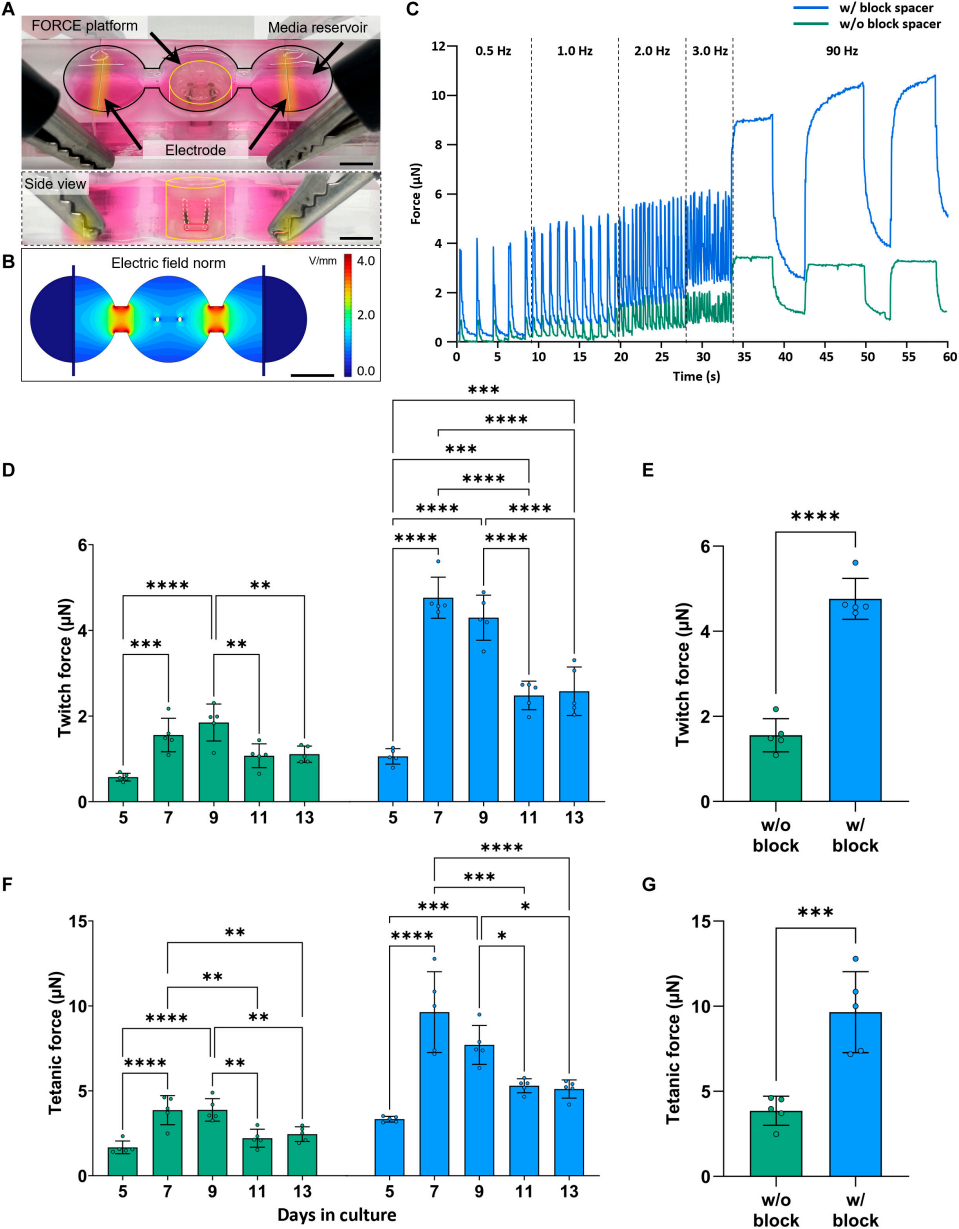
Quantification of enhanced contractile function in PDMS block-supplemented muscle tissue. (A) Photograph of the custom 3-well stimulation platform showing electrode placement in the side wells; inset, side view. Scale bar, 5 mm. (B) FEA of the electric-field distribution within the central observation well. Scale bar, 5 mm. (C) Real-time contraction traces of day-7 tissues stimulated at 0.5 to 90 Hz, comparing constructs matured with or without the PDMS spacer. (D and E) Twitch force (μN) across culture time points (D) and group comparison at day 7 (E) (*n* = 5). (F and G) Tetanic force (μN) across culture time points (F) and group comparison at day 7 (G) (*n* = 5). Asterisks in panels D to G indicate *P < 0.05, **P < 0.01, ***P < 0.001, and ****P < 0.0001.

We next asked whether spacer-induced improvements in alignment and differentiation translated into enhanced contractility. Tissues were stimulated electrically via a custom device sized for a 48-well plate with electrodes placed in the side wells (Fig. 6A and B and Fig. S5). We applied 0.5, 1.0, 2.0, 3.0, and 90 Hz to evoke twitch and tetanus. Real-time pillar deflection was tracked and converted to force using a calibrated spring constant *k* = 0.249 μN/μm obtained from mechanical testing of the pillar structure (Fig. 6C).

Contractile forces were minimal through day 5, peaked at day 7, and then declined by day 14. This trend occurred with and without the spacer, but absolute forces were consistently higher with the spacer (Fig. 6C to F). In addition, constructs formed from passage-8 cells exhibited inferior morphology and weaker contractility compared with passage-3 cells (Fig. S6 and Movies S2 to S4). Although C2C12 is an immortalized line, passage-dependent phenotypic drift has been reported and may reduce myogenic differentiation and contractile output, often attributed to culture-driven selection of subpopulations with altered myogenic propensity and shifts in myogenic programs [40,41]. Accordingly, passage numbers were controlled and matched across conditions to improve reproducibility [42].

These results demonstrate that maintaining a constant interpillar distance with the PDMS spacer improves not only alignment and myogenesis but also functional performance in contractility assays, consistent with structure–function coupling whereby alignment and tissue organization enhance force transmission and increase twitch/tetanic outputs [33]. The data further support pillar-based, displacement-driven assays as a valid readout of functional maturation for 3D muscle constructs.

Force peaked at day 7 and then declined in both groups; this pattern can be explained by 2 nonexclusive mechanisms. First, displacement-based calculations can underestimate force when additional stimulation-evoked deflection is small relative to pre-deflection (pre-tension). Correction by measuring and subtracting pre-tension is often required [15,33]. This is particularly relevant without the spacer, where interpillar distance decreased continuously to day 14 (Fig. 5G), implying rising passive tension. Notably, a similar decline occurred with the spacer, where interpillar distance during culture was fixed; upon spacer removal for measurement, however, internal pre-tension likely induced immediate shortening before acquisition—consistent with progressive post-removal shortening at later time points (Fig. S7)—which could skew force conversion.

Second, active force may have genuinely decreased due to suboptimal muscle length relative to the length–tension curve [43,44]. Excessive shortening (no-spacer condition) or overstretch (spacer condition) can reduce cross-bridge overlap and shift the balance toward passive tension as a protective response, diminishing active force.

Although additional experiments are required to adjudicate these mechanisms, the PDMS-spacer group consistently outperformed controls across formation, alignment, differentiation, and contractility. From a translational perspective, this architecture is not inherently limited to C2C12 cells, and prior studies support scalable phenotypic drug testing using engineered primary human skeletal muscle microtissues and human induced pluripotent-stem-cell-derived skeletal muscle microtissues in multiwell, pillar-based formats [14,32]. Moreover, its postpeak decline was slower, suggesting that spacer-assisted maturation stabilized tissue structure and function more effectively than culture without a controlled boundary condition.

### Drug-response evaluation using the FORCE platform

**Fig. 7.**
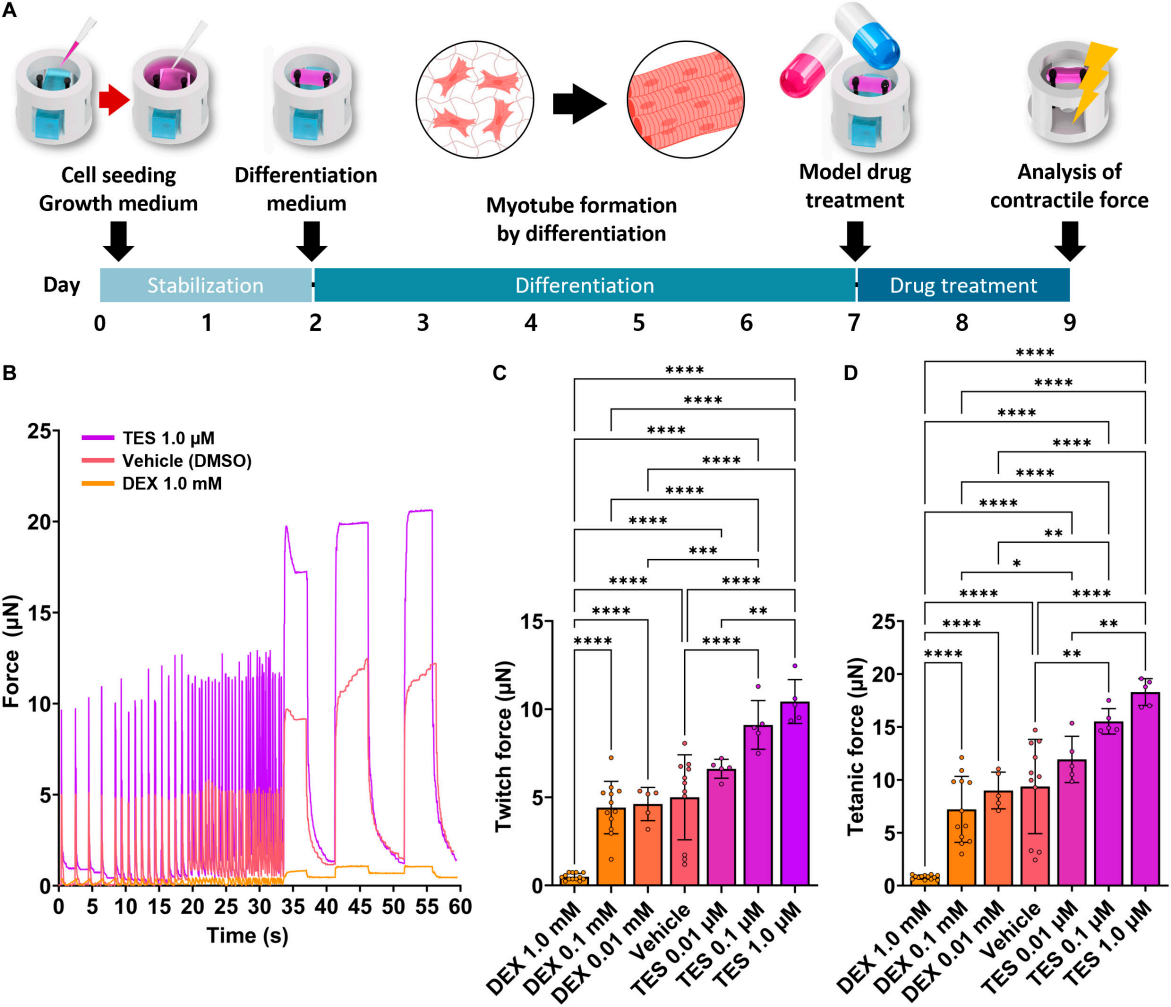
Drug-response evaluation of C2C12 engineered muscle tissues on the FORCE platform. (A) Experimental design workflow for tissue maturation and drug treatment. After C2C12 seeding and differentiation, engineered muscle tissues were treated from day 7 to day 9 (48 h) with dexamethasone (DEX; 0.01, 0.1, and 1.0 mM) or testosterone (TES; 0.01, 0.1, and 1.0 μM), followed by contractility analysis. (B) Real-time contraction traces of tissues stimulated at 0.5 to 90 Hz under EPS, showing drug-dependent changes in contraction dynamics. DMSO, dimethyl sulfoxide. (C) Quantification of twitch force (μN) across drug conditions, showing a dose-dependent decrease with DEX and an increasing trend with TES compared with vehicle (*n* ≥ 5). (D) Quantification of tetanic force (μN) across drug conditions, demonstrating a similar dose-dependent reduction with DEX and an increasing trend with TES (*n* ≥ 5). Asterisks in panels C and D indicate *P < 0.05, **P < 0.01, ***P < 0.001, and ****P < 0.0001.

To evaluate the utility of the FORCE platform for drug-response evaluation, we assessed its responsiveness to compounds with distinct pharmacological effects on skeletal muscle function. DEX is widely used to induce skeletal muscle atrophy and functional decline [45], whereas TES has been reported to promote myogenic differentiation and myotube hypertrophy through anabolic signaling and androgen-receptor-related pathways [46]. These 2 compounds were therefore selected as a contrasting pair for evaluating drug responsiveness on the platform. C2C12-based engineered muscle tissues were cultured and differentiated on the FORCE platform until day 7, followed by treatment with DEX (0.01, 0.1, and 1.0 mM) or TES (0.01, 0.1, and 1.0 μM) for 48 h from day 7 to day 9. Twitch and tetanic contractile forces were quantified after treatment (Fig. 7A).

Before contractility analysis, we assessed whether the selected drug-treatment conditions induced acute cytotoxicity. To this end, C2C12 cells were cultured under 2-dimensional conditions until day 7 and exposed to the same concentration range for 48 h. Under all tested conditions, including DEX, TES, and vehicle (DMSO), cell viability remained above 90%, indicating that the concentrations and treatment duration used in this study induced limited acute cell death (Fig. S8). Contractile function analysis on the FORCE platform showed that DEX treatment reduced both twitch and tetanic force in a dose-dependent manner (Fig. 7B to D). In particular, contractile force was markedly decreased at 1.0 mM DEX, and a decreasing tendency relative to the vehicle group was also observed at 0.1 mM. In contrast, TES-treated groups exhibited increased twitch and tetanic force, with significant increases observed at 0.1 and 1.0 μM compared with the vehicle group (Fig. 7B to D). Notably, the vehicle (DMSO) group was not significantly different from the untreated group in terms of contractile force, indicating that vehicle-related effects did not materially influence the interpretation of the results (Fig. S9A and B).

The FORCE platform used in this study also provides material advantages for drug-response evaluation. Whereas previously reported skeletal muscle-on-a-chip platforms have largely relied on PDMS-based soft lithography, PDMS-based microfluidic devices are known to reduce the effective concentration of small molecules because of absorption/adsorption-related loss [47]. In contrast, the FORCE platform was coated with parylene C, which forms a conformal thin film through chemical vapor deposition and is known to provide favorable barrier properties and chemical stability [48]. Accordingly, the parylene C coating may help reduce the influence of small-molecule absorption/adsorption and provide a more stable environment for evaluating dose-dependent responses.

The response patterns observed here were consistent with previous reports. DEX has been widely used to induce an atrophy-like phenotype in C2C12 myotubes [49], whereas TES has been reported to promote myogenic differentiation and myotube hypertrophy through androgen-receptor-related signaling pathways [46]. A similar tendency was also supported by immunofluorescence analysis, in which the TES 1.0-μM-treated group showed stronger MHC and F-actin signals than the DEX 1.0-mM- and vehicle (DMSO)-treated groups, consistent with the contractility results (Fig. S10) [12,50]. Taken together, these results suggest that the FORCE platform can quantitatively distinguish drug-induced changes in contractile function under the same force-readout conditions and may serve as a useful platform for evaluating dose-dependent drug responses.

## Conclusion

This study demonstrates a monolithic, 3D-printed FORCE platform supplemented with a removable PDMS block spacer for generating functional engineered skeletal muscle. The spacer imposes a fixed-length boundary condition, preventing excessive tissue shortening that typically undermines in vitro maturation. Across key metrics—tissue alignment, myotube differentiation, and contractile performance—constructs matured with the spacer outperformed controls. Notably, although contractile force peaked at day 7 in all groups, tissues cultured with the spacer showed a slower subsequent decline, consistent with more stable structural maturation under optimized mechanical loading. Together, these findings establish the PDMS-supplemented FORCE platform as a robust, reproducible tool for long-term muscle maturation studies and quantitative contractility assays, with direct applicability to tissue-engineering and drug-screening workflows.

## Data Availability

The datasets used and/or analyzed during the current study are available from the corresponding authors on reasonable request.

## References

[1] Cruz-Jentoft AJ, Bahat G, Bauer J, Boirie Y, Bruyère O, Cederholm T, Cooper C, Landi F, Rolland Y, Sayer AA. Sarcopenia: Revised European consensus on definition and diagnosis. Age Ageing. 2019;48(1):16–31. 30312372 10.1093/ageing/afy169 PMC6322506

[2] Stearns-Reider KM, D’Amore A, Beezhold K, Rothrauff B, Cavalli L, Wagner WR, Vorp DA, Tsamis A, Shinde S, Zhang C. Aging of the skeletal muscle extracellular matrix drives a stem cell fibrogenic conversion. Aging Cell. 2017;16(3):518–528. 28371268 10.1111/acel.12578 PMC5418187

[3] Urciuolo A, Serena E, Ghua R, Zatti S, Giomo M, Mattei N, Vetralla M, Selmin G, Luni C, Vitulo N. Engineering a 3D in vitro model of human skeletal muscle at the single fiber scale. PLOS ONE. 2020;15(5): Article e0232081. 32374763 10.1371/journal.pone.0232081 PMC7202609

[4] Schiaffino S, Reggiani C. Fiber types in mammalian skeletal muscles. Physiol Rev. 2011;91(4):1447–1531. 22013216 10.1152/physrev.00031.2010

[5] Egan B, Zierath JR. Exercise metabolism and the molecular regulation of skeletal muscle adaptation. Cell Metab. 2013;17(2):162–184. 23395166 10.1016/j.cmet.2012.12.012

[6] Zhao R, Chen CS, Reich DH. Force-driven evolution of mesoscale structure in engineered 3D microtissues and the modulation of tissue stiffening. Biomaterials. 2014;35(19):5056–5064. 24630092 10.1016/j.biomaterials.2014.02.020 PMC4046864

[7] Gillies AR, Lieber RL. Structure and function of the skeletal muscle extracellular matrix. Muscle Nerve. 2011;44(3):318–331. 21949456 10.1002/mus.22094 PMC3177172

[8] Truskey GA. Development and application of human skeletal muscle microphysiological systems. Lab Chip. 2018;18(20):3061–3073. 30183050 10.1039/c8lc00553b PMC6177290

[9] Hamaguchi H, Matsui TS, Deguchi S, Furuichi Y, Fujii NL, Manabe Y. Establishment of a system evaluating the contractile force of electrically stimulated myotubes from wrinkles formed on elastic substrate. Sci Rep. 2022;12(1):13818. 35970858 10.1038/s41598-022-17548-7 PMC9378739

[10] Santoso JW, Li X, Gupta D, Suh GC, Hendricks E, Lin S, Perry S, Ichida JK, Dickman D, McCain ML. Engineering skeletal muscle tissues with advanced maturity improves synapse formation with human induced pluripotent stem cell-derived motor neurons. APL Bioeng. 2021;5(3): Article 036101. 34286174 10.1063/5.0054984 PMC8282350

[11] Jangir H, Hickman JJ. Mimicking the tendon microenvironment to enhance skeletal muscle adhesion and longevity in a functional microcantilever platform. ACS Biomater Sci Eng. 2023;9(8):4698–4708. 37462389 10.1021/acsbiomaterials.3c00235 PMC10430766

[12] Khodabukus A, Kaza A, Wang J, Prabhu N, Goldstein R, Vaidya VS, Bursac N. Tissue-engineered human myobundle system as a platform for evaluation of skeletal muscle injury biomarkers. Toxicol Sci. 2020;176(1):124–136. 32294208 10.1093/toxsci/kfaa049 PMC7643536

[13] Akiyama Y, Nakayama A, Nakano S, Amiya R, Hirose J. An electrical stimulation culture system for daily maintenance-free muscle tissue production. Cyborg Bionic Syst. 2021;2021: Article 9820505. 36285137 10.34133/2021/9820505 PMC9494718

[14] Afshar ME, Abraha HY, Bakooshli MA, Davoudi S, Thavandiran N, Tung K, Ahn H, Ginsberg HJ, Zandstra PW, Gilbert PM. A 96-well culture platform enables longitudinal analyses of engineered human skeletal muscle microtissue strength. Sci Rep. 2020;10(1):6918. 32332853 10.1038/s41598-020-62837-8 PMC7181829

[15] Hofemeier AD, Limon T, Muenker TM, Wallmeyer B, Jurado A, Afshar ME, Ebrahimi M, Tsukanov R, Oleksiievets N, Enderlein J. Global and local tension measurements in biomimetic skeletal muscle tissues reveals early mechanical homeostasis. eLife. 2021;10: Article e60145. 33459593 10.7554/eLife.60145 PMC7906603

[16] van der Wal E, Iuliano A, In ’t Groen SLM, Bholasing AP, Priesmann D, Sharma P, den Hamer B, Saggiomo V, Krüger M, Pijnappel WP. Highly contractile 3D tissue engineered skeletal muscles from human iPSCs reveal similarities with primary myoblast-derived tissues. Stem Cell Rep. 2023;18(10):1954–1971. 10.1016/j.stemcr.2023.08.014 PMC10656354 37774701

[17] Legant WR, Pathak A, Yang MT, Deshpande VS, McMeeking RM, Chen CS. Microfabricated tissue gauges to measure and manipulate forces from 3D microtissues. Proc Natl Acad Sci USA. 2009;106(25):10097–10102. 19541627 10.1073/pnas.0900174106 PMC2700905

[18] Boudou T, Legant WR, Mu A, Borochin MA, Thavandiran N, Radisic M, Zandstra PW, Epstein JA, Margulies KB, Chen CS. A microfabricated platform to measure and manipulate the mechanics of engineered cardiac microtissues. Tissue Eng Part A. 2012;18(9–10):910–919. 22092279 10.1089/ten.tea.2011.0341 PMC3338105

[19] Rodriguez ML, Werner TR, Becker B, Eschenhagen T, Hirt MN. Magnetics-based approach for fine-tuning afterload in engineered heart tissues. ACS Biomater Sci Eng. 2019;5(7):3663–3675. 31637285 10.1021/acsbiomaterials.8b01568 PMC6803165

[20] Walker M, Rizzuto P, Godin M, Pelling AE. Structural and mechanical remodeling of the cytoskeleton maintains tensional homeostasis in 3D microtissues under acute dynamic stretch. Sci Rep. 2020;10(1):7696. 32376876 10.1038/s41598-020-64725-7 PMC7203149

[21] Leonard A, Bertero A, Powers JD, Beussman KM, Bhandari S, Regnier M, Murry CE, Sniadecki NJ. Afterload promotes maturation of human induced pluripotent stem cell derived cardiomyocytes in engineered heart tissues. J Mol Cell Cardiol. 2018;118:147–158. 29604261 10.1016/j.yjmcc.2018.03.016 PMC5940558

[22] Xu Y, Qi J, Zhou W, Liu X, Zhang L, Yao X, Wu H. Generation of ring-shaped human iPSC-derived functional heart microtissues in a Möbius strip configuration. Bio-Des Manuf. 2022;5(4):687–699.

[23] Ong LJY, Kasetsirikul S, Milton LA, Nie X, Eiken AW, Chirnside J, Young LMG, Nordin GP, Toh YC. 3D-printed unibody microfluidic devices for organ-on-chip applications. Adv Mater Technol. 2025;10(22): Article e01056.

[24] Kim J, Lee ZF, Sim GD, Jeon JS. Strategic approaches in generation of robust microphysiological 3D musculoskeletal tissue system. Adv Funct Mater. 2024;34(52):2410872.

[25] Kah D, Lell J, Wach T, Spörrer M, Dessalles CA, Wiedenmann S, Gerum RC, Vergarajauregui SL, Esser TU, Böhringer D. Contractility of cardiac and skeletal muscle tissue increases with environmental stiffness. bioRxiv. 2024. https://www.biorxiv.org/content/10.1101/2024.02.23.581737v1.full

[26] Morita T, Nie M, Takeuchi S. Tetanus-driven biohybrid multijoint robots powered by muscle rings with enhanced contractile force. Sci Adv. 2025;11(29): Article eadu9962. 40668910 10.1126/sciadv.adu9962 PMC12266100

[27] Jo B, Motoi K, Morimoto Y, Takeuchi S. Dynamic and static workout of in vitro skeletal muscle tissue through a weight training device. Adv Healthc Mater. 2024;13(32):2401844. 39212188 10.1002/adhm.202401844 PMC11670278

[28] Xavier MS, Tawk CD, Yong YK, Fleming AJ. 3D-printed omnidirectional soft pneumatic actuators: Design, modeling and characterization. Sens Actuators A Phys. 2021;332: Article 113199.

[29] Musgrove HB, Cook SR, Pompano RR. Parylene-C coating protects resin-3D-printed devices from material erosion and prevents cytotoxicity toward primary cells. ACS Appl Bio Mater. 2023;6(8):3079–3083. 10.1021/acsabm.3c00444 PMC10754061 37534979

[30] Fernández-Costa JM, Ortega MA, Rodríguez-Comas J, Lopez-Muñoz G, Yeste J, Mangas-Florencio L, Fernández-González M, Martin-Lasierra E, Tejedera-Villafranca A, Ramon-Azcon J. Training-on-a-chip: A multi-organ device to study the effect of muscle exercise on insulin secretion in vitro. Adv Mater Technol. 2023;8(7):2200873.

[31] Kim J, Hur SS, Park JH, Ban MJ, Kim JY, Kim JH, Choo JH, Kim HW, Lee JH, Lee MR. Cell surface thiol engineering mechanoregulates myogenic differentiation via the FAK–PI3K–AKT axis. Adv Healthc Mater. 2025; Article e00914. 40787751 10.1002/adhm.202500914 PMC12805617

[32] Iuliano A, Haalstra M, Raghuraman R, Bielawski K, Bholasing AP, van der Wal E, de Greef JC, Pijnappel WP. Real-time and multichannel measurement of contractility of hiPSC-derived 3D skeletal muscle using fiber optics-based sensing. Adv Mater Technol 2023;8(22):2300845.

[33] Lewis PL, Yan M, Su J, Shah RN. Directing the growth and alignment of biliary epithelium within extracellular matrix hydrogels. Acta Biomater. 2019;85:84–93. 30590182 10.1016/j.actbio.2018.12.039 PMC6768828

[34] Danesini PC, Heim M, Tomalka A, Siebert T, Ates F. Endomysium determines active and passive force production in muscle fibers. J Biomech. 2024;168: Article 112134. 38723428 10.1016/j.jbiomech.2024.112134

[35] Schätzlein E, Blaeser A. Recent trends in bioartificial muscle engineering and their applications in cultured meat, biorobotic systems and biohybrid implants. Commun Biol. 2022;5(1):737. 35869250 10.1038/s42003-022-03593-5 PMC9307618

[36] Noonan AM, Mashouri P, Chen J, Power GA, Brown SH. Training induced changes to skeletal muscle passive properties are evident in both single fibers and fiber bundles in the rat hindlimb. Front Physiol. 2020;11:907. 32903515 10.3389/fphys.2020.00907 PMC7435064

[37] Sleboda DA, Wold ES, Roberts TJ. Passive muscle tension increases in proportion to intramuscular fluid volume. J Exp Biol. 2019;222(21): Article jeb209668. 31558592 10.1242/jeb.209668 PMC6857584

[38] Vesga-Castro C, Aldazabal J, Vallejo-Illarramendi A, Paredes J. Contractile force assessment methods for in vitro skeletal muscle tissues. eLife. 2022;11: Article e77204. 35604384 10.7554/eLife.77204 PMC9126583

[39] Dostanić M, Wiendels M, Windt LM, Mol MP, van den Hil FE, Davis RP, Orlova V, van Meer BJ, Mastrangeli M, Mummery CL. Tapered pillar design for high-precision force readout in miniaturized engineered heart tissues from human pluripotent stem cells. Adv Healthc Mater. 2025;14(31): Article e01664. 40884127 10.1002/adhm.202501664 PMC12683227

[40] Asakura A, Kikyo N. Immunofluorescence analysis of myogenic differentiation. *Methods Cell Biol*. 2022;170:117–125. 10.1016/bs.mcb.2022.02.010 PMC9699006 35811095

[41] Elashry MI, Kinde M, Klymiuk MC, Eldaey A, Wenisch S, Arnhold S. The effect of hypoxia on myogenic differentiation and multipotency of the skeletal muscle-derived stem cells in mice. Stem Cell Res Ther. 2022;13(1):56. 35123554 10.1186/s13287-022-02730-5 PMC8817503

[42] Taye N, Stanley S, Hubmacher D. Stable knockdown of genes encoding extracellular matrix proteins in the C2C12 myoblast cell line using small-hairpin (sh)RNA. J Vis Exp. 2020;(156): 10.3791/60824. 10.3791/60824 PMC7553128 32116296

[43] Pham S, Puckett Y. Physiology, skeletal muscle contraction. Treasure Island (FL): StatPearls Publishing; 2025. 32644432

[44] Lieber RL, Ward SR. Skeletal muscle design to meet functional demands. Philos Trans R Soc Lond B Biol Sci. 2011;366(1570):1466–1476. 21502118 10.1098/rstb.2010.0316 PMC3130443

[45] Ulla A, Uchida T, Miki Y, Sugiura K, Higashitani A, Kobayashi T, Ohno A, Nakao R, Hirasaka K, Sakakibara I. Morin attenuates dexamethasone-mediated oxidative stress and atrophy in mouse C2C12 skeletal myotubes. Arch Biochem Biophys. 2021;704: Article 108873. 33848514 10.1016/j.abb.2021.108873

[46] Wannenes F, Caprio M, Gatta L, Fabbri A, Bonini S, Moretti C. Androgen receptor expression during C2C12 skeletal muscle cell line differentiation. Mol Cell Endocrinol. 2008;292(1–2):11–19. 18588941 10.1016/j.mce.2008.05.018

[47] Sasaki H, Onoe H, Osaki T, Kawano R, Takeuchi S. Parylene-coating in PDMS microfluidic channels prevents the absorption of fluorescent dyes. Sens Actuators B Chem. 2010;150(1):478–482.

[48] Cherukuri R, Kim S, Moyer HL, Jesse H, Lam PY, Richardson LS, Kammala AK, Menon R, Rusyn I, Han A. Reducing small molecule adsorption in a PDMS-based microphysiological system of the female reproductive tract via Parylene-C coating to improve mechanistic studies. ACS Appl Mater Interfaces. 2026;18(2):3565–3577. 41493331 10.1021/acsami.5c20917 PMC12828717

[49] Yoshioka K, Ito A, Arifuzzaman M, Yoshigai T, Fan F, Sato K-i, Shimizu K, Kawabe Y, Kamihira M. Miniaturized skeletal muscle tissue fabrication for measuring contractile activity. J Biosci Bioeng. 2021;131(4):434–441. 33358352 10.1016/j.jbiosc.2020.11.014

[50] Ito A, Yamamoto Y, Sato M, Ikeda K, Yamamoto M, Fujita H, Nagamori E, Kawabe Y, Kamihira M. Induction of functional tissue-engineered skeletal muscle constructs by defined electrical stimulation. Sci Rep. 2014;4(1):4781. 24759171 10.1038/srep04781 PMC3998029

