# Supplementary material for: A Monolithic 3D-Printed Platform for Functional Maturation and In Situ Contractility Assessment of 3D Skeletal Muscle
Source: Biomater Res. 2026 May 13;30:0363. doi: 10.34133/bmr.0363 (PMC13168760; doi:10.34133/bmr.0363)
Supplement: Supplementary 1 — Figs. S1 to S10 Tables S1 to S3 Movies S1 to S4 [file bmr.0363.f1.zip › 2_Revised Supplementary materials.docx]

SUPPLEMENTARY MATERIALS


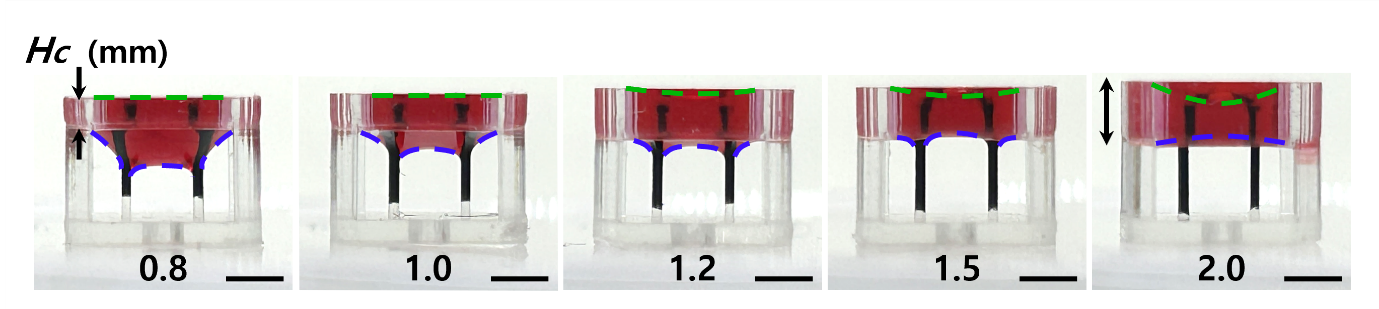


**Figure S1.** **Suspended-channel height (*H*c) governs patterned hydrogel thickness and profile.** Macroscale side-view images of patterned hydrogel loaded into channels of varying *H*c. Green and blue dotted lines denote the top and bottom hydrogel boundaries, respectively, illustrating the height-dependent change in the meniscus-induced sagitta (surface depth). Scale bar, 2 mm.


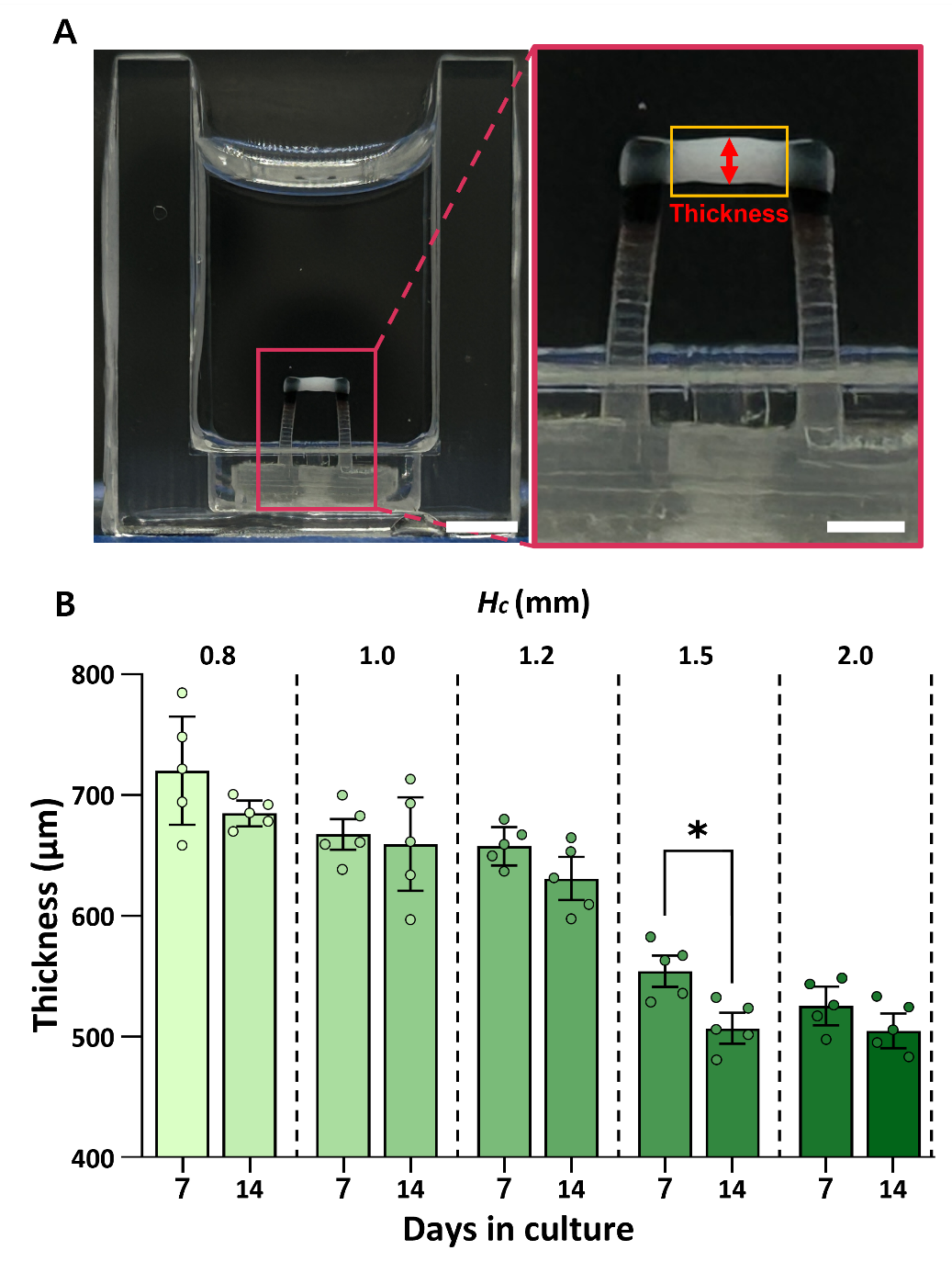


**Figure S2.** **Suspended-channel height (*H*c) modulates tissue thickness over culture time.** (A) Representative side-view images of muscle constructs after removal of the suspended-channel frame from the FORCE platform. Scale bars, left 3 mm; right 1 mm. (B) Quantitative comparison of tissue thickness at day 7 and day 14 across *H*c conditions. A statistically significant thickness reduction from day 7 to day 14 was observed specifically at *H*c = 1.5 mm (n = 5).


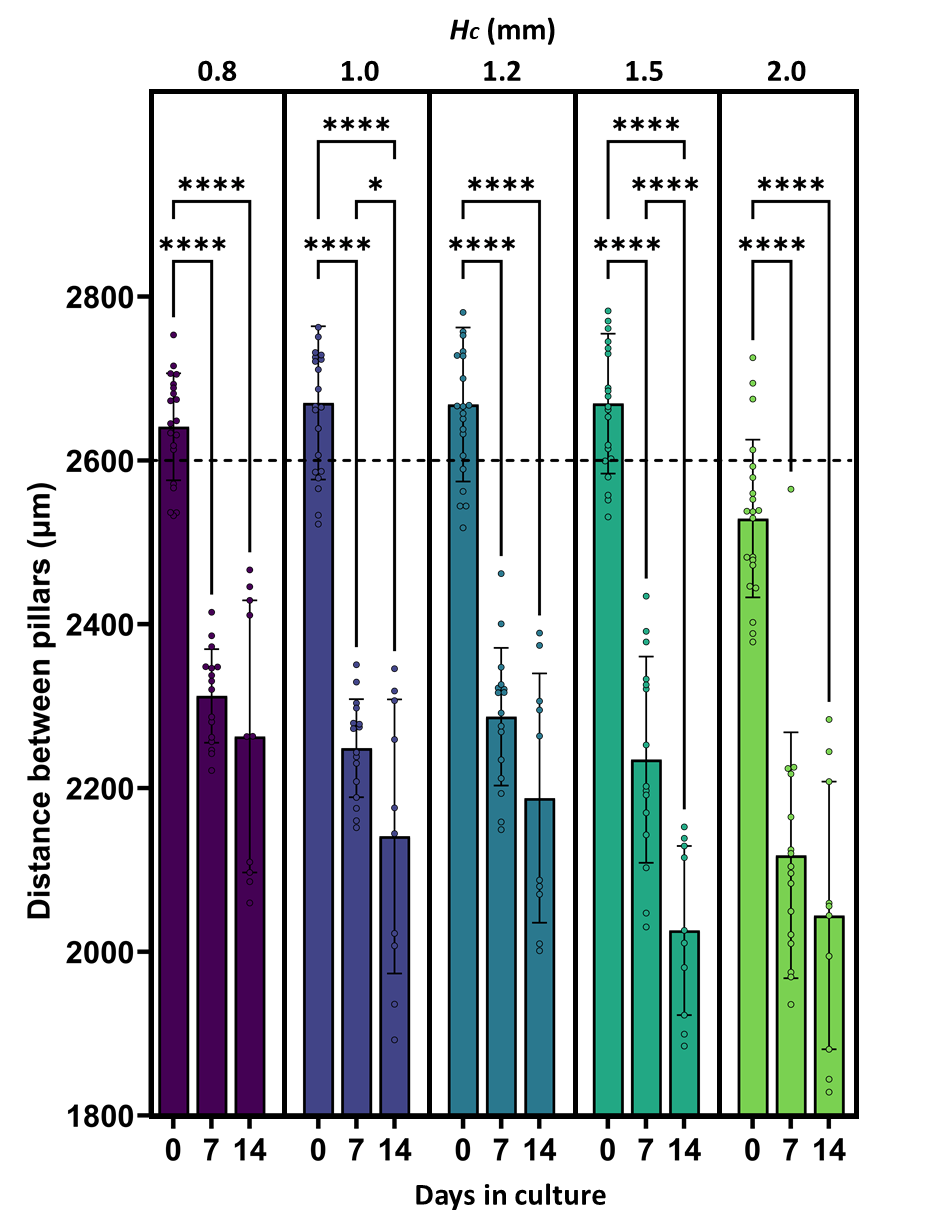


**Figure S3.** **Suspended-channel height (*H*c)–dependent trajectories of interpillar distance over 14 days.** Interpillar distance (μm) across the 14-day culture period for each *H*c condition. Data are replotted from Figure 3F to enable within-condition comparisons at day 0, day 7, and day 14 (n ≥ 10).


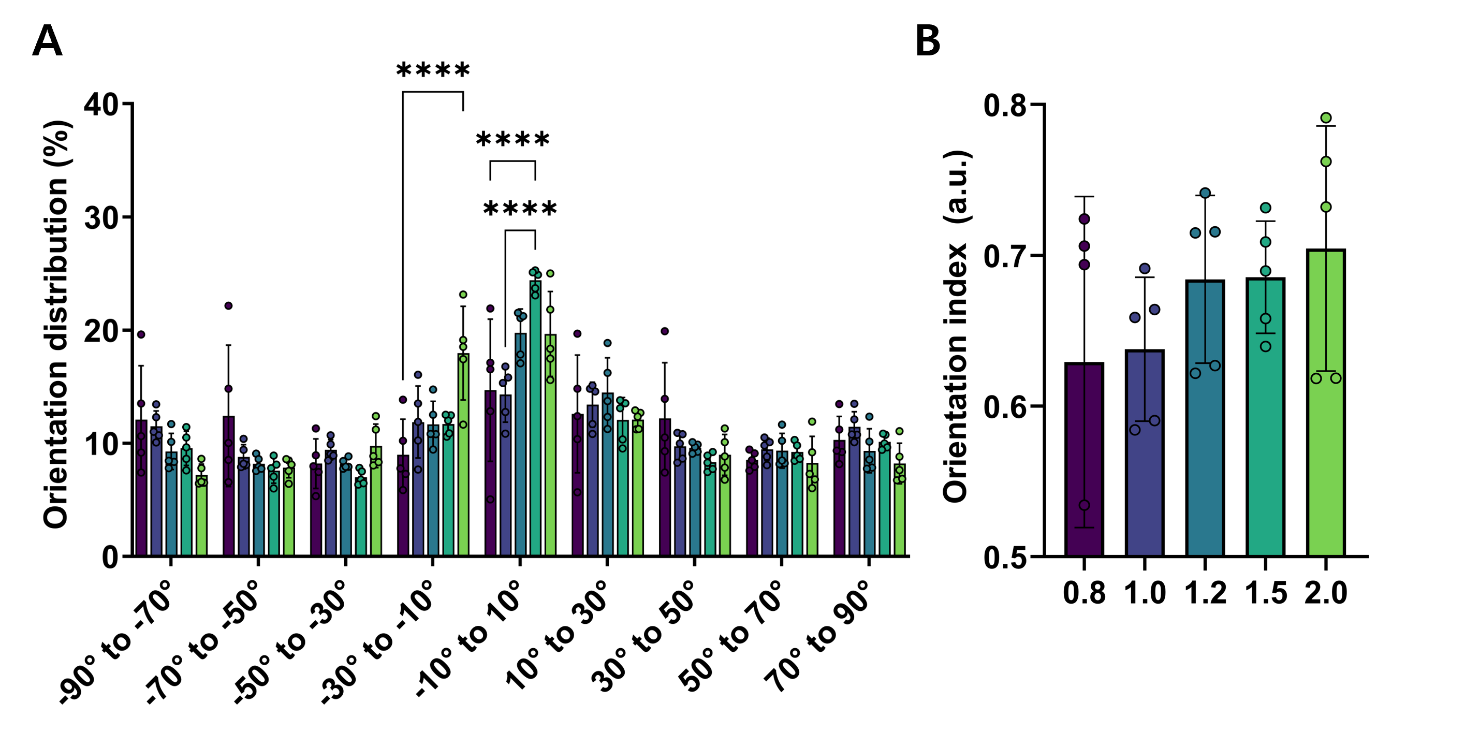


**Figure S4.** **Myofiber alignment at day 7 as a function of suspended-channel height (*H*c).** (A) Myofiber orientation distribution maps at day 7 for each *H*c condition (n = 5). (B) Orientation Index (OI) quantification comparing alignment across *H*c at day 7 (n = 5).


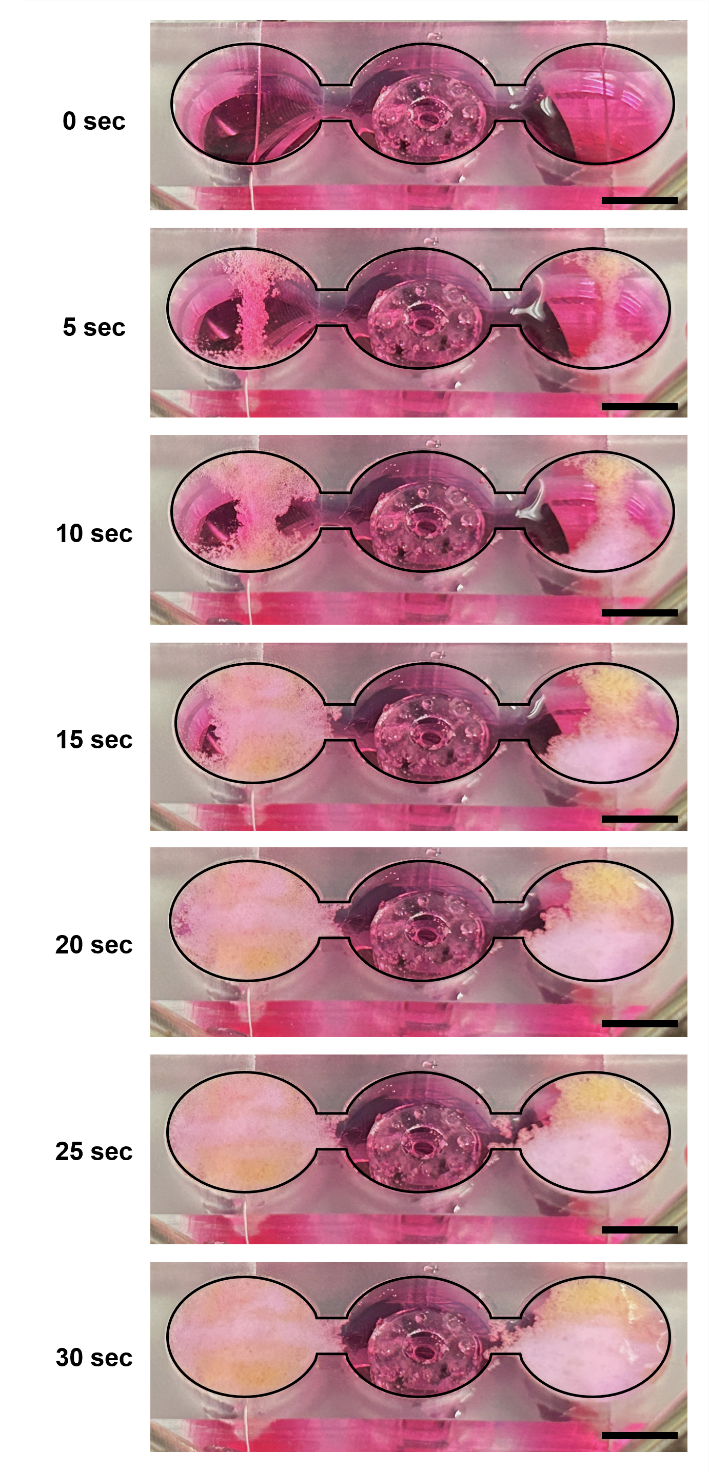


**Figure S5.** **Compartmentalization of gas bubbles during electrical pulse stimulation (EPS).** Time-lapse images (0–30 s) of the custom three-well module under continuous EPS (40 V, 10 ms pulse width, 90 Hz) with electrodes placed in the side wells. Bubbles remain localized near the electrodes and do not enter the central observation well for ≤30 s, defining a 30-s single-run stimulation window. Scale bar, 5 mm.


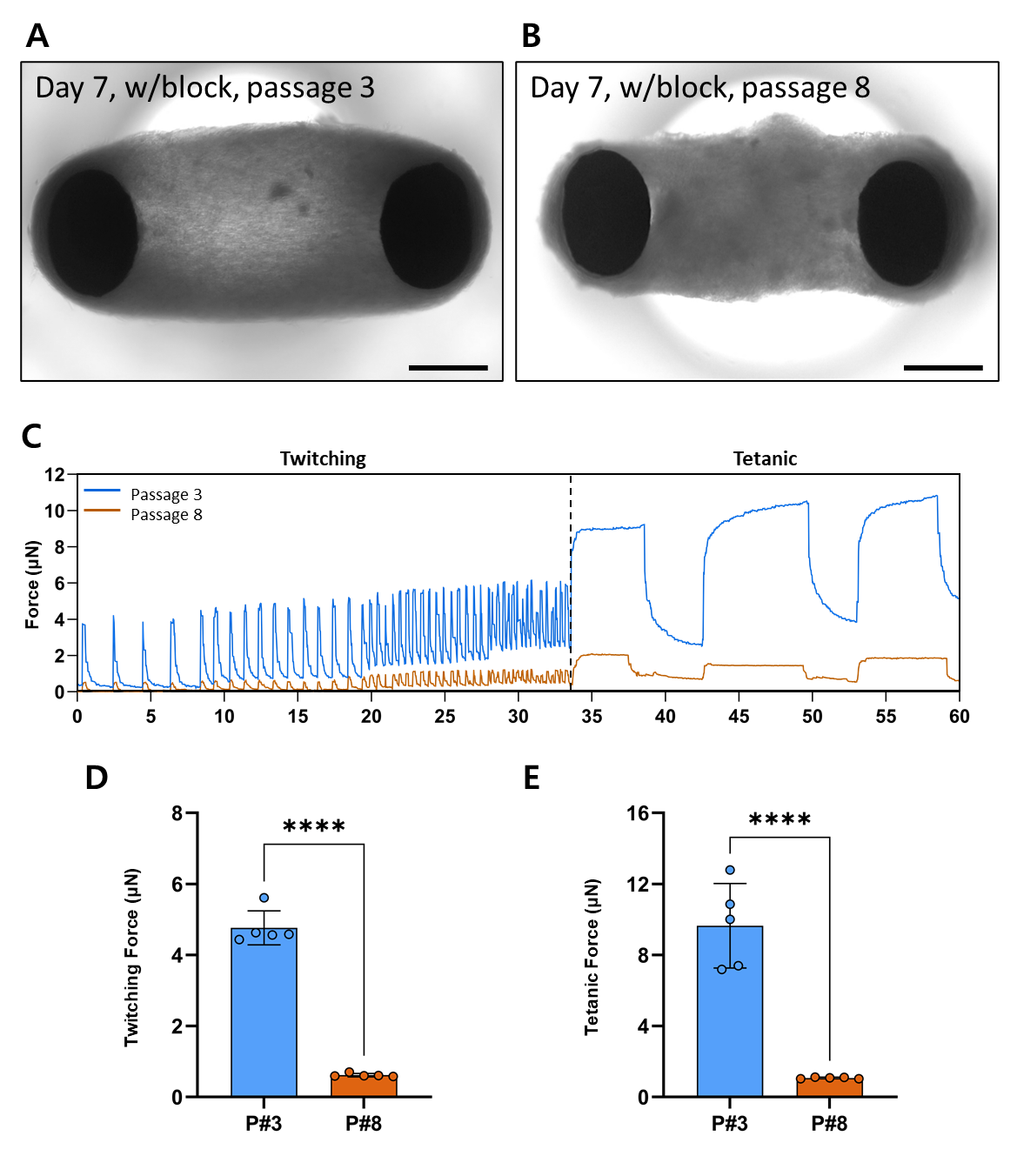


**Figure S6. Passage number affects engineered muscle morphology and contractility**. (A, B) Day-7 bright-field images of constructs matured with the PDMS (polydimethylsiloxane) spacer using passage-3 (P3; A) and passage-8 (P8; B) C2C12 cells. Scale bar, 500 μm. (C) Real-time contraction traces for P3 and P8 tissues under electrical stimulation. (D, E) Quantification of twitch force (D) and tetanic force (E) (μN) comparing P3 and P8, demonstrating significantly reduced contractility at higher passage (n = 5).


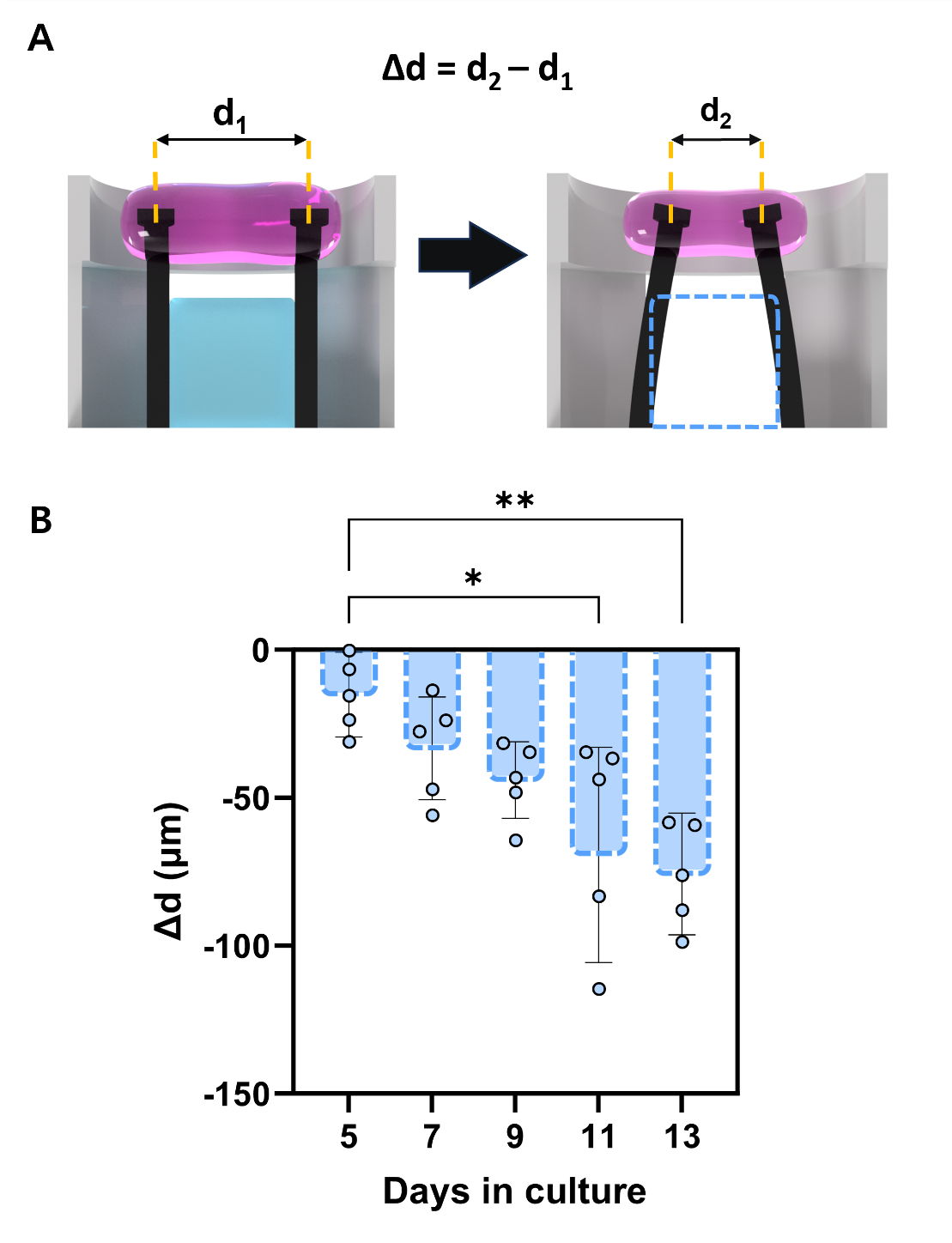


**Figure S7. Progressive passive tension inferred from interpillar distance change (Δd) after PDMS spacer removal.** (A) Schematic defining the change in interpillar distance, Δd = d_2_ - d_1_, where d_1_ was measured immediately before PDMS spacer removal and d_2_ was measured immediately after removal. Negative Δd indicates immediate shortening. (B) Quantification of Δd (µm) immediately after spacer removal as a function of culture time (days 5–13), (n = 5).


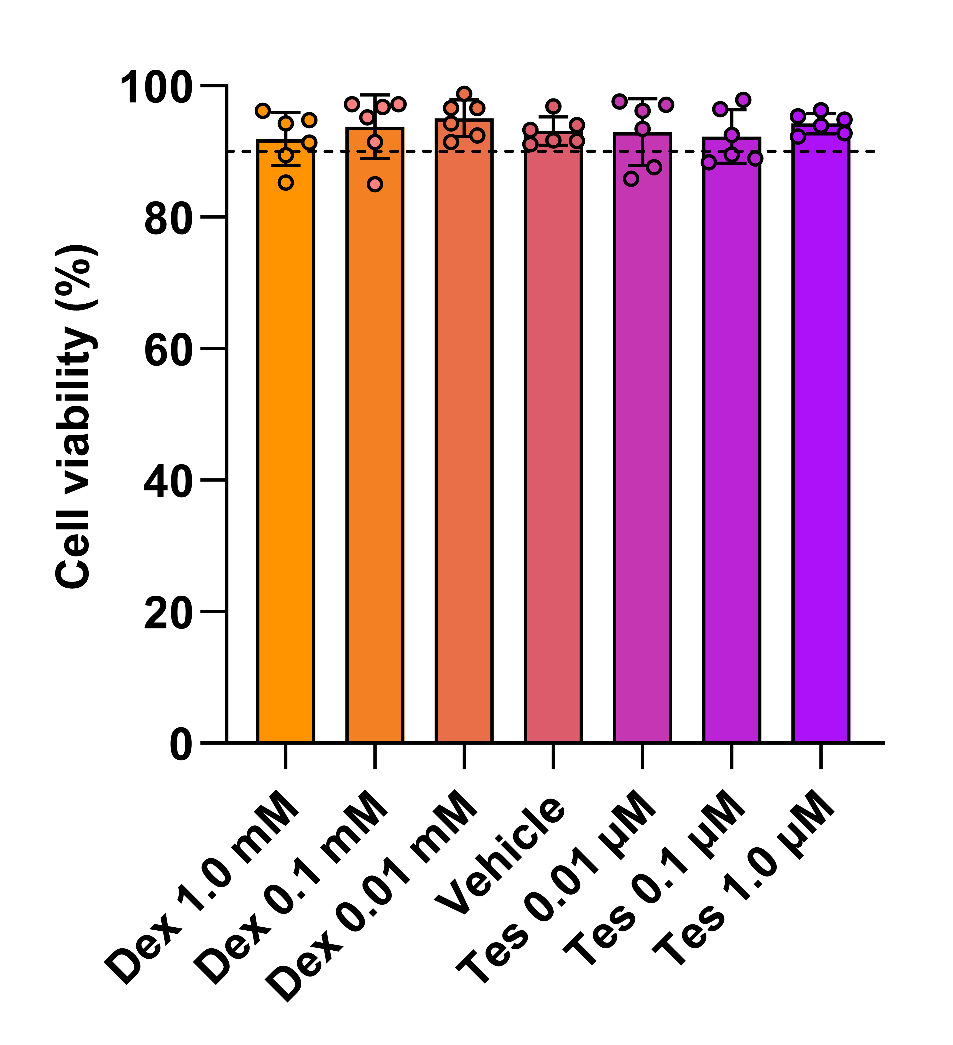


**Figure S8. Cell viability after dose-dependent treatment with testosterone, dexamethasone, and vehicle (DMSO).** C2C12 cells cultured under 2D conditions to day 7 were treated with TES, DEX, or DMSO at the indicated concentrations for 48 h, and viability was quantified using a live/dead assay (n = 6).


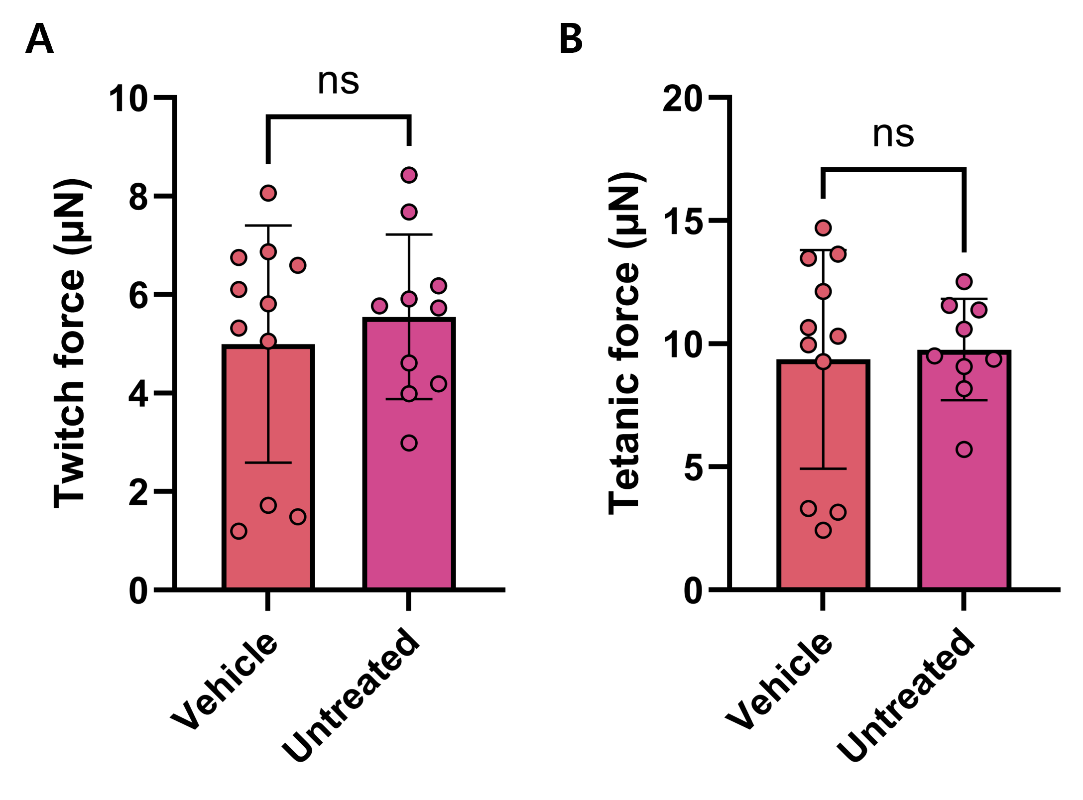


**Figure S9. Comparison of contractile forces with and without vehicle (DMSO) treatment in FORCE-cultured engineered muscle tissues.** (A) Twitch force in DMSO-treated tissues compared with untreated controls. (B) Tetanic force in DMSO-treated tissues compared with untreated controls (n ≥ 10).


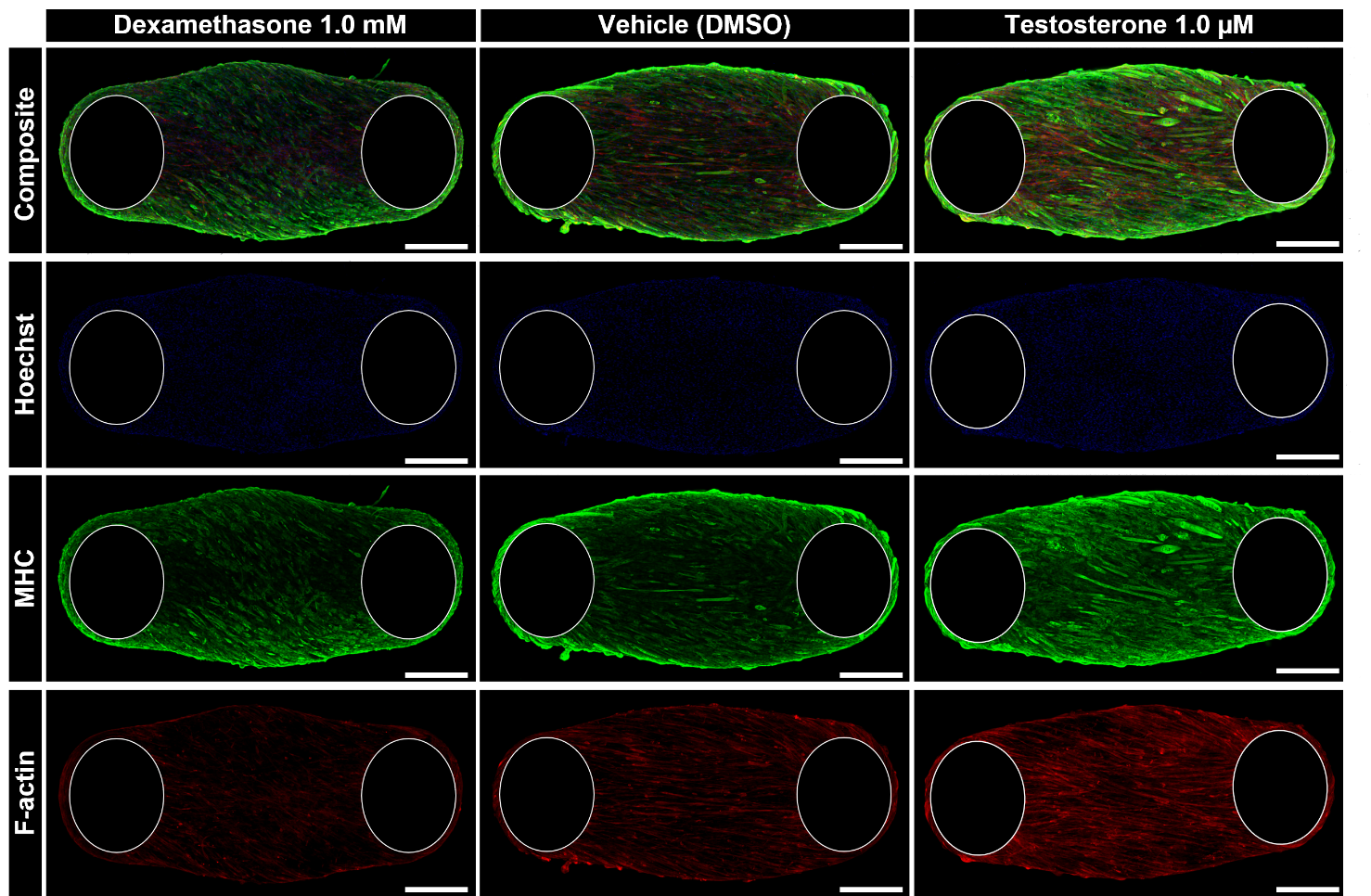


**Figure S10. Immunofluorescence imaging of engineered muscle tissues following drug treatment.** Engineered muscle tissues formed on the FORCE platform were treated for 48 h with TES, DEX or DMSO, followed by immunofluorescence staining. Representative images show nuclei (Hoechst, blue), myosin heavy chain (MHC, green), F-actin (red), and merged composites. Scale bar, 500 µm.

| Figure | Groups levels | N | Test statistic | *p*-value | Effect size (η²) |
| --- | --- | --- | --- | --- | --- |
| Fig. 2D | 5 | 60 | F(4, 55)= 43.60 | <0.0001 | 0.760 |
| Fig. 2E | 4 | 237 | F(3, 233)= 169.7 | <0.0001 | 0.686 |
| Fig. 2F | 4 | 265 | F(3, 261)= 43.44 | <0.0001 | 0.333 |
| Fig. 3B | 5 | 25 | F(4, 20)= 140.4 | <0.0001 | 0.9656 |
| Fig. 3E | 5 | 25 | F(4, 20)= 28.67 | <0.0001 | 0.8515 |
| Fig. 3G-i | 5 | 25 | F(4, 20)= 7.832 | 0.0006 | 0.610 |
| Fig. 5D | 5 (w/o block spacer) | 25 | F(4, 20)= 13.00 | <0.0001 | 0.7222 |
| Fig. 5D | 5 (w/ block spacer) | 25 | F(4, 20)= 57.88 | <0.0001 | 0.9205 |
| Fig. 5F | 5 (w/o block spacer) | 25 | F(4, 20)= 14.18 | <0.0001 | 0.7393 |
| Fig. 5F | 5 (w/ block spacer) | 26 | F(4, 20)= 20.38 | <0.0001 | 0.8030 |
| Fig. 6C | 7 | 56 | F(6, 49)= 41.19 | <0.0001 | 0.8345 |
| Fig. 6D | 7 | 56 | F(6, 49)= 37.10 | <0.0001 | 0.8196 |
| Fig. S3 | 3 (Group 0.8) | 46 | F(2, 43)= 78.57 | <0.0001 | 0.7851 |
| Fig. S3 | 3 (Group 1.0) | 47 | F(2, 44)= 117.2 | <0.0001 | 0.8420 |
| Fig. S3 | 3 (Group 1.2) | 47 | F(2, 44)= 94.12 | <0.0001 | 0.8105 |
| Fig. S3 | 3 (Group 1.5) | 46 | F(2, 43)= 154.2 | <0.0001 | 0.8776 |
| Fig. S3 | 3 (Group 2.0) | 47 | F(2, 44)= 65.62 | <0.0001 | 0.7489 |
| Fig. S4B | 5 | 25 | F(4, 20)= 1.062 | 0.4008 | 0.1753 |
| Fig. S7B | 5 | 25 | F(4, 20)= 6.510 | 0.0016 | 0.5656 |
| Fig. S8 | 7 | 42 | F(6, 35)= 0.5270 | 0.7839 | 0.08286 |

**Table S1. Statistical summary of one-way ANOVA.** Statistical outcomes for all one-way ANOVA analyses, including F statistics with degrees of freedom, exact two-sided p-values, and effect size (η²). Where applicable, post hoc multiple-comparison tests with multiplicity correction are reported to identify pairwise group differences.

| Figure | Row levels | Column levels | Effect | Test statistic | *p*-value | Effect size (η²) |
| --- | --- | --- | --- | --- | --- | --- |
| Fig. 3F | 3 | 5 | Interaction | F(8, 218)= 2.786 | 0.0059 | 0.0176 |
|  |  |  | Row factor | F(2, 218)= 480.3 | <0.0001 | 0.7573 |
|  |  |  | Column factor | F(4, 218)= 17.13 | <0.0001 | 0.0540 |
| Fig. 3G | 9 | 5 | Interaction | F(32, 180)= 6.092 | <0.0001 | 0.3056 |
|  |  |  | Row factor | F(8, 180)= 32.88 | <0.0001 | 0.4123 |
|  |  |  | Column factor | F(4, 180)= 1.359e-9 | >0.9999 | 8.519e-12 |
| Fig. 4G | 3 | 2 | Interaction | F(2, 69)= 16.61 | <0.0001 | 0.0674 |
|  |  |  | Row factor | F(2, 69)= 153.5 | <0.0001 | 0.6228 |
|  |  |  | Column factor | F(1, 69)= 66.63 | <0.0001 | 0.1352 |
| Fig. 4I | 9 | 2 | Interaction | F(8, 72)= 19.87 | <0.0001 | 0.08199 |
|  |  |  | Row factor | F(8, 72)= 213.4 | <0.0001 | 0.8807 |
|  |  |  | Column factor | F(1, 72)= 0.3313 | 0.5667 | 1.709e-4 |
| Fig. 4K | 9 | 2 | Interaction | F(8, 72)= 16.28 | <0.0001 | 0.1124 |
|  |  |  | Row factor | F(8, 72)= 119.6 | <0.0001 | 0.8254 |
|  |  |  | Column factor | F(1, 72)= 0.07249 | 0.7885 | 6.256e-5 |
| Fig. S2B | 5 | 2 | Interaction | F(4, 40)= 1.068 | 0.3848 | 0.0070 |
|  |  |  | Row factor | F(4, 40)= 135.7 | <0.0001 | 0.8962 |
|  |  |  | Column factor | F(1, 40)= 18.60 | <0.0001 | 0.0307 |
| Fig. S4A | 9 | 5 | Interaction | F(32, 180)= 4.138 | <0.0001 | 0.2102 |
|  |  |  | Row factor | F(8, 180)= 39.71 | <0.0001 | 0.5041 |
|  |  |  | Column factor | F(4, 180)= 0.00048 | >0.9999 | 3.101e-6 |

**Table S2. Statistical summary of two-way ANOVA.** Statistical outcomes for all two-way ANOVA analyses are summarized, including F statistics with degrees of freedom, exact two-sided P values, and effect size (η²) for the interaction term and each main effect. Where applicable, post hoc multiple-comparison tests with multiplicity correction are reported to identify specific pairwise differences across factor levels.

| Figure | Groups levels | N | Test statistic | p-value | Cohen’s d |
| --- | --- | --- | --- | --- | --- |
| Fig. 1D | 2 (24h) | 10 | t(8)= 30.19 | <0.0001 | 19.10 |
| Fig. 1D | 2 (48h) | 10 | t(8)= 54.70 | <0.0001 | 34.60 |
| Fig. 1D | 2 (72h) | 10 | t(8)= 26.71 | <0.0001 | 16.89 |
| Fig. 4H | 2 | 10 | t(8)= 6.746 | 0.0001 | 4.27 |
| Fig. 4I-i | 2 | 10 | t(8)= 8.608 | <0.0001 | 5.44 |
| Fig. 4J | 2 | 10 | t(8)= 7.469 | <0.0001 | 4.72 |
| Fig. 4K-i | 2 | 10 | t(8)= 7.540 | <0.0001 | 4.77 |
| Fig. 5E | 2 | 10 | t(8)= 11.58 | <0.0001 | 7.32 |
| Fig. 5G | 2 | 10 | t(8)= 5.116 | 0.0009 | 3.24 |
| Fig. S6D | 2 | 10 | t(8)= 19.23 | <0.0001 | 12.16 |
| Fig. S6E | 2 | 10 | t(8)= 8.057 | <0.0001 | 5.10 |
| Fig. S9A | 2 | 21 | t(19)= 0.6013 | 0.5547 | 0.263 |
| Fig. S9B | 2 | 20 | t(18)= 0.2452 | 0.8091 | 0.110 |

**Table S3.** **Statistical summary of unpaired t-tests.** Statistical outcomes for all unpaired two-tailed t-tests are summarized, including sample size (N), t statistics with degrees of freedom, exact two-sided P values, and effect size (Cohen’s d) for each comparison. Statistical significance was assessed at P < 0.05.

**Movie S1.** Representative video showing the removal process of the PDMS block spacer.

**Movie S2.** Representative video of contracting in vitro skeletal muscle tissue cultured with a block spacer on the FORCE platform (Day 7, Passage 3).

**Movie S3.** Representative video of contracting in vitro skeletal muscle tissue cultured without a block spacer on the FORCE platform (Day 7, Passage 3).

**Movie S4.** Representative video of contracting in vitro skeletal muscle tissue cultured without a block spacer on the FORCE platform (Day 7, Passage 8).
